# Dihomo-γ-linolenic acid inhibits several key cellular processes associated with atherosclerosis

**DOI:** 10.1016/j.bbadis.2019.06.011

**Published:** 2019-09-01

**Authors:** Hayley Gallagher, Jessica O. Williams, Nele Ferekidis, Alaa Ismail, Yee-Hung Chan, Daryn R. Michael, Irina A. Guschina, Victoria J. Tyrrell, Valerie B. O'Donnell, John L. Harwood, Inna Khozin-Goldberg, Sammy Boussiba, Dipak P. Ramji

**Affiliations:** a Cardiff School of Biosciences, Cardiff University, Sir Martin Evans Building, Museum Avenue, Cardiff CF10 3AX, UK; b Systems Immunity Research Institute, School of Medicine, Cardiff University, Cardiff CF14 4XN, UK; c Microalgal Biotechnology Laboratory, French Associates Institute for Agriculture and Biotechnology of Drylands, J. Blaustein Institutes for Desert Research, Ben-Gurion University of the Negev, Sede Boqer Campus, 84990, Israel

**Keywords:** ABC, ATP-binding cassette transporter, AcLDL, acetylated LDL, Apo, apolipoprotein, CE, cholesteryl esters, CVD, cardiovascular disease, DGLA, dihomo-γ-linolenic acid, ECM, extracellular matrix, FC, free cholesterol, FCCP, carbonyl cyanide-*4*-(trifluoromethoxy)phenylhydrazone, HASMC, human aortic smooth muscle cells, GLA, gamma-linolenic acid, HMDM, human monocyte-derived macrophages, HUVEC, human umbilical cord endothelial cells, ICAM-1, intercellular adhesion molecule-1, IFN-γ, interferon-γ, IL, interleukin, LA, linoleic acid, LPS, lipopolysaccharide, LXR, liver X receptors, LY, lucifer yellow, MCP-1, monocyte chemotactic protein-1, NEFA, non esterified fatty acids, oxLDL, oxidized LDL, PBMC, peripheral blood mononuclear cells, PDGF, platelet-derived growth factor, PGE1, prostaglandin E1, PUFA, polyunsaturated fatty acid, RT-qPCR, real-time quantitative polymerase chain reaction, SR, scavenger receptor, STAT-1, signal transducer and activator of transcription-1, TBHP, tert-butyl hydroperoxide, TNF-α, tumour necrosis factor-α, VCAM-1, vascular cell adhesion molecule-1, VSMC, vascular smooth muscle cells, Atherosclerosis, Dihomo-γ-linolenic acid, Foam cells, Inflammation, Macrophages, Smooth muscle cells, Gene expression

## Abstract

Atherosclerosis and its complications are responsible for one in three global deaths. Nutraceuticals show promise in the prevention and treatment of atherosclerosis but require an indepth understanding of the mechanisms underlying their actions. A previous study showed that the omega-6 fatty acid, dihomo-γ-linolenic acid (DGLA), attenuated atherosclerosis in the apolipoprotein E deficient mouse model system. However, the mechanisms underlying such protective effects of DGLA are poorly understood and were therefore investigated. We show that DGLA attenuates chemokine-driven monocytic migration together with foam cell formation and the expression of key pro-atherogenic genes induced by three pro-inflammatory cytokines in human macrophages. The effect of DGLA on interferon-γ signaling was mediated via inhibition of signal transducer and activator of transcription-1 phosphorylation on serine 727. In relation to anti-foam cell action, DGLA inhibits modified LDL uptake by both macropinocytosis and receptor-mediated endocytosis, the latter by reduction in expression of two key scavenger receptors (SR-A and CD36), and stimulates cholesterol efflux from foam cells. DGLA also improves macrophage mitochondrial bioenergetic profile by decreasing proton leak. Gamma-linolenic acid and prostaglandin E1, upstream precursor and key metabolite respectively of DGLA, also acted in an anti-atherogenic manner. The actions of DGLA extended to other key atherosclerosis-associated cell types with attenuation of endothelial cell proliferation and migration of smooth muscle cells in response to platelet-derived growth factor. This study provides novel insights into the molecular mechanisms underlying the anti-atherogenic actions of DGLA and supports further assessments on its protective effects on plaque regression *in vivo* and in human trials.

## Highlights

• Dihomo-γ-linolenic acid (DGLA) attenuates atherosclerosis in a mouse model system.
• The mechanisms underlying anti-atherogenic actions of DGLA are poorly understood.
• DGLA inhibited atherogenic processes in three key cell types in this disease.
• Mechanisms underlying such protective actions of DGLA were identified.
• Studies inform on the beneficial anti-atherogenic actions of DGLA.

## 1 Introduction

Atherosclerosis, an inflammatory disease of the vasculature and the underlying cause of cardiovascular disease (CVD), is responsible for about 31.5% of all global deaths [[1], [2], [3]]. The disease represents a major healthcare and economic burden and this may worsen in the future because of global increases in risk factors such as diabetes and obesity [1,2]. Atherosclerosis is initiated in response to various risk factors, particularly the accumulation of LDL in the intima of arteries and its subsequent oxidation to oxidized LDL (oxLDL) [[1], [2], [3]]. This then triggers arterial endothelial cell activation and/or dysfunction leading to secretion of chemokines by these cells and increased expression of adhesion proteins on their cell surface [[1], [2], [3]]. Immune cells, particularly monocytes, are recruited to the site of oxLDL accumulation [[1], [2], [3]]. These monocytes differentiate into macrophages that then take up oxLDL by various processes, including scavenger receptor (SR)-mediated endocytosis and macropinocytosis, that together with defective cholesterol efflux from these cells results in the formation of lipid-laden foam cells [3,4]. As the disease progresses, foam cells lyse by apoptosis and necrosis leading to the formation of a lipid-rich necrotic core associated with a chronic inflammatory response orchestrated by cytokines such as interferon-γ (IFN-γ), tumour necrosis factor-α (TNF-α) and interleukin (IL)-1β [5,6]. Smooth muscle cells proliferate and migrate from the media to the intima to stabilize the plaques by the production of an extracellular matrix (ECM) that forms part of the fibrous cap of atherosclerotic lesions [[2], [3], [4], [5]]. The stability of such plaques is dictated in part by the synthesis and degradation of the ECM [[2], [3], [4], [5]]. Excessive degradation by a range of proteases produced under inflammatory conditions causes plaque destabilization and ultimately rupture and subsequent thrombosis [[2], [3], [4], [5]].

Statins have had significant impact in the recent reduction of CVD morbidity and mortality in developed countries [2,7]. However, many clinical trials have highlighted the considerable residual risk of CVD in patients on statin therapy together with issues such as tolerability and patient-dependent efficacy [2,7]. Emerging pharmaceutical therapies such as ezetimibe, which inhibits the dietary absorption of cholesterol, monoclonal antibodies or small interfering RNA that target the protease proprotein convertase subtilisin/kexin type 9 involved in the degradation of the LDL receptor, and monoclonal antibodies against IL-1β have shown some promise though many of these are expensive [2]. Unfortunately, the recent clinical outcomes on numerous pharmaceutical agents against established targets (e.g. inhibitors of cholesteryl ester transfer protein, acyl-coenzyme A acyltransferase 1 and lipoprotein-associated phospholipase A_2_) have been disappointing [2,8]. Nutraceuticals (food products with health benefits beyond their nutritional value) represent promising agents for the prevention of atherosclerosis and as add-on with current therapies but require an in-depth understanding of their beneficial effects together with the underlying mechanisms of their actions *in vitro* and *in vivo* [2,3].

Polyunsaturated fatty acids (PUFAs) have many health benefits and previous research on omega-3 PUFAs has demonstrated several anti-atherogenic actions [1,2,9]. Some omega-6 PUFAs also have health benefits that are poorly understood because of a general paucity of research performed on them in comparison to omega-3 PUFAs or many other nutraceuticals [1,2,9]. Further studies on beneficial omega-6 PUFAs are required to inform on their use as preventative and therapeutic agents and because of issues of sustainability and environment associated with the use of omega-3-rich fish oils [9]. DGLA is an important omega-6 PUFA that is well tolerated with no side effects in studies on animal model systems and in humans [[10], [11], [12]]. Limited studies have shown association of low levels of DGLA with ischemic heart disease and severity of coronary artery disease together with risk factors such as insulin resistance, non-insulin dependent diabetes mellitus and metabolic syndrome [[13], [14], [15]]. In contrast, DGLA levels are higher in Eskimos who have low prevalence of coronary artery disease [16]. DGLA also reversed hypertension in rats by diets rich in saturated fats [17], decreased cutaneous inflammatory responses in mice produced by croton oil [18] and demonstrated antithrombotic potential in humans [19]. In addition, studies on apolipoprotein E deficient mice (ApoE^−/−^) fed a normal chow diet supplemented with DGLA for 6 months showed reduced lipid deposition in the aorta together with the content of macrophages and smooth muscle cells and the expression of adhesion molecules intercellular adhesion molecule-1 (ICAM-1) and vascular cell adhesion molecule-1 (VCAM-1) [20]. Furthermore, in ApoE^−/−^ mice fed a Paigen diet for 1 month, DGLA decreased areas of lipid accumulation in the aorta together with the expression of adhesion proteins [20]. Although providing evidence for an anti-atherogenic role of DGLA, this study was rather restrictive in terms of mechanistic insight and provided no indications on the effects of DGLA on several key cellular processes associated with atherosclerosis. We have therefore investigated this key aspect with particular focus on macrophages, a key cell type involved in all the different stages of the disease [21]. The studies were also extended to some key atherosclerosis-associated processes in endothelial cells and vascular smooth muscle cells (VSMC).

## 2 Materials and methods

### 2.1 Reagents

Human THP-1 cell line, human umbilical cord endothelial cells (HUVEC) and human aortic smooth muscle cells (HASMC) together with murine RAW264.7 cell line were from Sigma-Aldrich. The other reagents were from: Abcam [lactate dehydrogenase (LDH) kit]; Cell Signaling Technology [anti phospho-STAT1 Tyr^701^ (91675), anti phospho-STAT1 Ser^727^ (91775)]; GE Healthcare (^14^C-cholesterol); Nu-Chek Prep, Inc. (DGLA); Cayman Chemical [prostaglandin E1 (PGE1) and gamma-linolenic acid (GLA)]; Nycomed Pharmaceuticals (Lymphoprep™); Peprotech [monocyte chemotactic protein-1 (MCP-1), IL-1β, IFN-γ, TNF-α]; Perkin-Elmer (1-^14^C-acetate); VWR Lifescience (Ribozol™); Santa Cruz Biotechnology Inc. [anti-STAT1 (Sc-592), anti-β-actin (Sc-130656), anti-SR-A (Sc-20660)] or Intracel [1,1′-dioctadecyl-3,3,3′,3′-tetramethyllindocarbocyane perchlorate (DiI)-labeled oxLDL (DiI-oxLDL) and acetylated LDL (AcLDL)].

### 2.2 Cell culture

Primary human monocyte-derived macrophages (HMDM) were isolated from monocytes obtained from buffy coats (National Blood Service Wales) using Ficoll-Hypaque purification as previously described [[22], [23], [24], [25]]. Ethical approval and informed consent for each donor was granted by the Welsh Blood Service for use of human blood samples. The investigation conforms to the principles outlined in the Declaration of Helsinki. Human THP-1 cell line, mouse RAW264.7 cell line and HMDM were cultured in RPMI1640 medium with stable glutamine supplemented with 10% (v/v) heat-inactivated foetal calf serum (HI-FCS), penicillin (100 U/ml) and streptomycin (100 μg/ml) at 37°C in a humidified atmosphere containing 5% (v/v) CO_2_. THP-1 monocytes were differentiated into macrophages by incubation for 24 h with 0.16 μM of phorbol 12-myristate 13-acetate (PMA) [6,[22], [23], [24], [25]]. HUVEC and HASMC were cultured in their appropriate ready to use media according to the manufacturer's instructions (Sigma-Aldrich). Cell viability and proliferation assays were carried out as our previous study [23].

### 2.3 Real-time quantitative PCR (RT-qPCR)

RNA isolation, reverse transcription and RT-qPCR analysis of resulting cDNA were performed as described elsewhere [6,22,23]. The sequences of the primers are given in Supplementary Table 1. The comparative ΔΔC_t_ method was used to represent relative expression normalized to the levels of the house-keeping gene [6,23].

### 2.4 Western blotting

Equal amounts of proteins were size-fractionated by SDS-PAGE alongside comparative molecular weight size markers to determine the size of the protein product and subjected to Western blot analysis as described elsewhere [6,22,24,25].

### 2.5 Migration assays

Monocyte migration in response to the chemokine MCP-1 was carried out as our previous study [23]. Migration of HASMC (1 × 10^5^ cells) was performed using a modified Boyden chamber with 8 μm porous insert coated with Matrigel [26] (E6909; Sigma-Aldrich). HASMC incubated with vehicle or DGLA for the requisite time in serum-free DMEM were in the upper chamber whereas the lower chamber contained 20 ng/ml platelet-derived growth factor (PDGF)-BB as a chemoattractant. After 4 h at 37°C in a humidified incubator with 5% (v/v) CO_2_, the inserts were carefully cut out using a scalpel and the cells on the underside of the membrane stained with Fluoroshield™ with DAPI mounting medium (F6057; Sigma-Aldrich). The membranes were visualised using fluorescent microscopy and the total number of cells in five different fields counted.

### 2.6 Analysis of parameters related to foam cell formation

Cells (2 × 10^6^) were pre-treated for 24 h with vehicle or DGLA in RPMI supplemented with 0.2% (v/v) fatty acid free BSA and then incubated in the absence or the presence of 50 μg/ml AcLDL and 1 μCi of [1-^14^C]-acetate for a further 24 h. The cells were scraped, pelleted by centrifugation at 9000 ×*g* and resuspended in 1 ml of distilled water. An aliquot was removed for protein analysis using a Micro BCA™ Protein assay Kit in accordance to the manufacturer's instructions (Thermo Fisher Scientific). To 1 ml of lysed cell suspension, 2 ml of chloroform: methanol (1:2, v/v) solution was added, mixed thoroughly and incubated for 15 min at room temperature. Then, 1 ml of chloroform and 2 ml of Garbus solution (2 M KCl in 0.5 M potassium phosphate buffer pH 7.6) [27] were added, mixed and centrifuged at 220 ×*g* for 3 min at room temperature. The chloroform layer containing the extracted lipids was dried in a stream of nitrogen, dissolved in 50 μl chloroform and used for the separation of polar lipids (PL) and non-polar lipids, including triacylglycerols (TAG), non-esterified fatty acids (NEFA), free cholesterol (FC) and cholesteryl esters (CE), by one dimension thin layer chromatography (TLC) on silica gel G plates (10 × 10 cm; Merk KGaA) using hexane: diethyl ether: acetic acid (80:20:1, v/v/v) solvent mixture. Radioactivity in the various lipid classes was determined by scintillation counting. Dil-oxLDL uptake, lucifer yellow (LY) uptake and cholesterol efflux assays were carried out as our previous studies [22,24,25,28].

### 2.7 Determination of PGE1 levels in cell supernatants

Hexane: isopropanol: 1 M acetic acid (30:20:2, v/v/v) was added to media collected from 4 × 10^6^ cells in a ratio of 2.5 ml of solvent mixture to 1 ml of media. An internal standard containing 10 ng PGE1-d4 was also included. Samples were mixed thoroughly by vortexing and 2.5 ml hexane was added followed by further vortexing. The samples were subjected to centrifugation (900 ×*g* for 5 min at room temperature) and the upper hexane layer transferred into a new glass tube. The hexane extraction was repeated again as above followed by evaporation of the samples under a stream of nitrogen and reconstitution in 200 μl of 100% HPLC grade methanol. The extracted lipids were then separated on a Spherisorb C_18_ ODS2, 5 μm, 150 mm × 4.6 mm column (Waters Ltd) using a gradient of 20–90% mobile phase B (A: water: acetonitrile: acetic acid, 75:25:0.1, v/v/v; B: methanol: acetonitrile: acetic acid, 60:40:0.1, v/v/v) over 60 min with a flow rate of 1 ml/min. PGE1 was quantified by LC-MS/MS electrospray ionization on a Q-Trap (Applied Biosystems 4000 Q-Trap) with specific multiple reaction monitoring (MRM) transitions (M−H^−^; PGE1: 353.2/273.2, PGE1-d4: 357.2/277.2) monitored as parent fragmenting to daughter with collisions energies of −20 to −28 V (MS conditions were DP −65, CE −30, dwell time 150 ms).

### 2.8 Determination of mitochondrial bioenergetic profile

This was determined using XF^e^96 Seahorse analyzer and XF cell mito stress test kit (mitochondrial respiration) together with XF-FluxPaks containing 96-well plates and cartridges (Agilent Technologies). Oligomycin (ATP synthase blocker), carbonyl cyanide-*4*-(trifluoromethoxy)phenylhydrazone (FCCP; mitochondrial uncoupler) and a mixture of rotenone (inhibitor of complex I) and antimycin-A (blocker of complex III) in the kit were serially injected to determine ATP-linked respiration, maximal respiration and non-mitrochondrial respiration respectively. These parameters together with basal respiration were then used to determine proton leak, spare respiratory capacity and coupling efficiency. Optimization experiments with different cell numbers and titration of various inhibitors were first carried out to assess various parameters as previous studies [29,30]. These were found to be 200,000 cells per well of a Seahorse plate, 1 μM oligomycin, 2 μM of FCCP and 0.15 μM rotenone/antimycin-A mix. A non-cell blank was included in all experiments. The experiments and data analysis were performed as described by the manufacturer (Agilent Technologies).

### 2.9 Statistical analysis

Data are presented as mean ± SEM on assigned number of independent experiments. Normality of data was tested using the Shapiro-Wilk test and single comparisons were performed using an unpaired Student's *t*-test. A One-way ANOVA with Tukey's post hoc analysis or Kruskal-Wallis test with Dunn's post hoc test were used when more than two groups were compared. The results were regarded as significant when p ≤ 0.05.

## 3 Results

### 3.1 DGLA attenuates pro-inflammatory gene expression in human and mouse macrophages

**Fig. 1:**
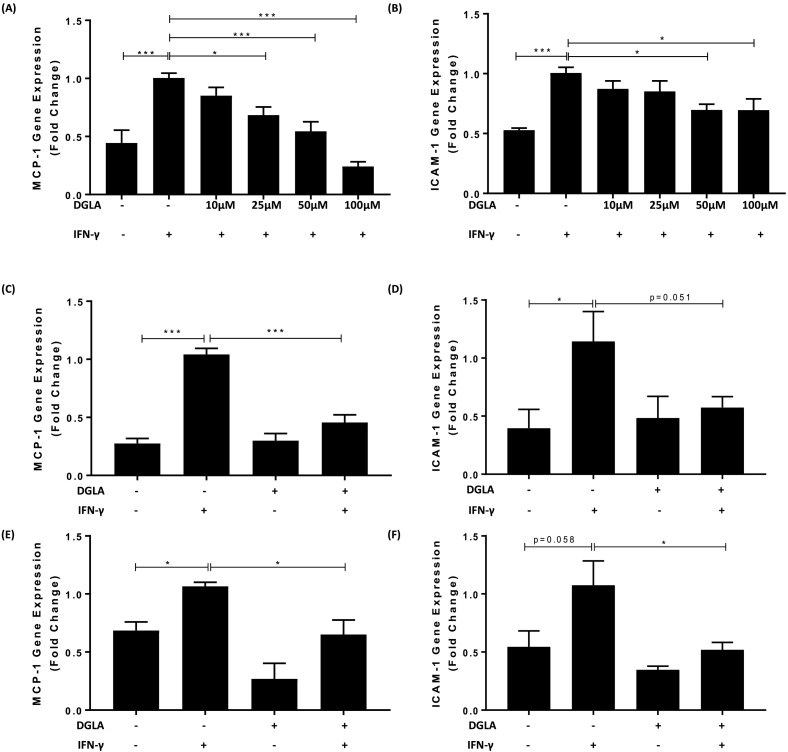
DGLA attenuates IFN-γ induced MCP-1 and ICAM-1 expression in human and mouse macrophages. THP-1 macrophages (A–B), primary HMDM (C–D) or mouse RAW264.7 macrophages (E–F) were pre-incubated for 24 h with the indicated concentrations of DGLA (A–B) or 50 μM DGLA (+, C–F) or DMSO vehicle (−). The cells were then incubated for 3 h with 250 U/ml IFN-γ (+) or its vehicle (−). Total RNA was subjected to RT-qPCR using primers against MCP-1 (A, C, E), ICAM-1 (B, D, F) or the housekeeping genes [human GAPDH (A–D) or mouse β-actin (E–F)]. The mRNA levels were calculated using the comparative Ct method and normalized to the housekeeping gene with values from cells pre-incubated with DMSO vehicle and then treated with IFN-γ given an arbitrary value of 1. Graphs display normalized gene expression (mean ± SEM) from three independent experiments. Statistical analysis was performed using a One-way ANOVA (equal variances) with Tukey's post hoc analysis (*, p ≤ 0.05; ***, p ≤ 0.001).

Previous studies showed that DGLA at concentrations of 50–200 μM inhibited lipopolysaccharide (LPS) induced TNF-α production in peripheral blood mononuclear cells (PBMC) [31]. To investigate whether such anti-inflammatory action extends to macrophages and other pro-inflammatory/atherogenic mediators, the effect of several concentrations of DGLA on IFN-γ induced expression of MCP-1 and ICAM-1 mRNA, which are both expressed at high levels during inflammation and atherosclerosis [5,23], was first investigated in human THP-1 macrophages. IFN-γ is a key pro-atherogenic cytokine and potentially one of the master regulator of the disease [32]. The human THP-1 cell line is used widely for studies of human monocytes/macrophages in atherosclerosis with demonstrated conservation of responses to primary cultures and *in vivo* [[22], [23], [24], [25],^33^,^34^]. As expected, the expression of both MCP-1 and ICAM-1 was significantly induced by IFN-γ (p < 0.001 in both cases; Fig. 1A–B). DGLA at concentrations of 25 μM, 50 μM and 100 μM produced significant inhibition of the IFN-γ-induced MCP-1 expression (p = 0.012, p < 0.001 and p < 0.001 respectively; Fig. 1A). For ICAM-1 a significant inhibition of IFN-γ-induced expression was seen with 50 μM and 100 μM DGLA (p = 0.050 and p = 0.038 respectively; Fig. 1B). Given the significant inhibition of IFN-γ-induced expression of two key pro-inflammatory genes by 50 μM DGLA, this concentration was used for subsequent studies unless or otherwise stated. Indeed, this concentration had no effect on the viability of cells, as judged by the release of the LDH enzyme, or their proliferation, as gauged by staining with crystal violet, both in the absence or the presence of IFN-γ stimulation (Supplementary Fig. 1). In addition, the use of 50 μM and 100 μM DGLA showed that the fatty acid was indeed taken up by the cells and incorporated into total polar lipid and triacylglycerol fractions (see C20:3n6 in Supplementary Fig. 2).

To rule out the possibility that the DGLA-mediated inhibition of IFN-γ induced MCP-1 and ICAM-1 expression was peculiar to the THP-1 cell line, comparative analysis was performed in primary HMDM. DGLA attenuates IFN-γ induced expression of MCP-1 and ICAM-1 in HMDM (p < 0.001 and p = 0.051 respectively; Fig. 1C–D). DGLA also inhibits the IFN-γ induced expression of MCP-1 and ICAM-1 in murine RAW264.7 macrophages (p = 0.022 and p = 0.045 respectively; Fig. 1E–F), thereby demonstrating conservation of response between human and mouse macrophages.

**Fig. 2:**
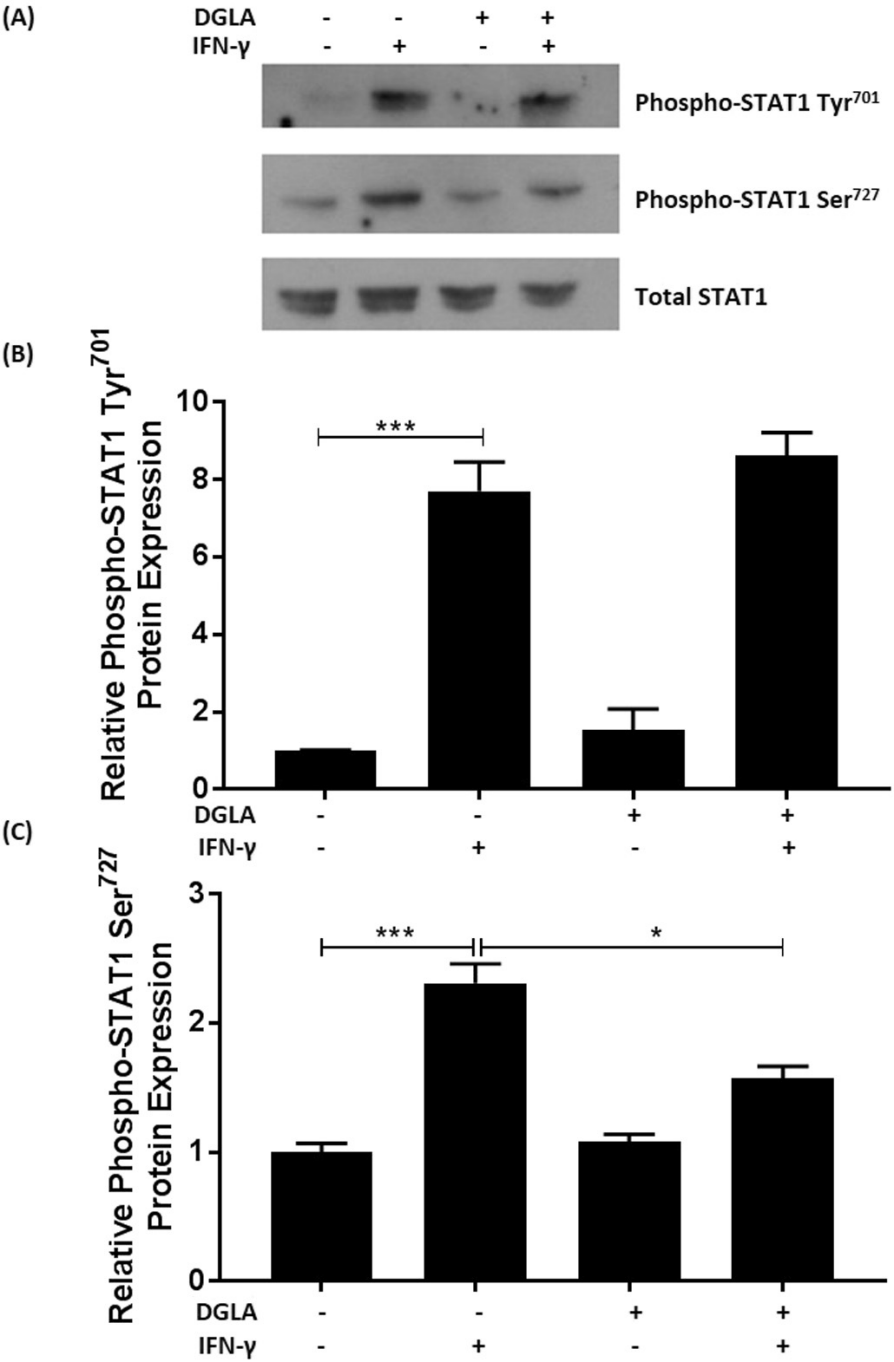
DGLA attenuates IFN-γ induced phosphorylation of STAT1 on serine 727. THP-1 macrophages were pre-treated with 50 μM DGLA (+) or DMSO vehicle (−) for 24 h. The cells were then stimulated for 30 min [24] with 250 U/ml IFN-γ (+) or its vehicle (−). Equal amount of proteins were subjected to Western blot analysis with antibodies against phospho-STAT1 Tyr^701^, phospho-STAT1 Ser^727^ or total STAT1. The data were subjected to densitometric and statistical analysis. A representative image is shown in (A) with graphs indicating the relative phosphorylation (mean ± SEM) on tyrosine^701^ (B) or serine^727^ (C) from three independent experiments. The values from cells treated with vehicle alone have been arbitrarily assigned as 1. Statistical analysis was performed using a One-way ANOVA with Tukey's post hoc analysis (*, p ≤ 0.05; ***, p ≤ 0.001).

Signal transducer and activator of transcription-1 (STAT-1) is a key transcription factor in IFN-γ signaling and involved in the inducible expression of the MCP-1 and ICAM-1 genes [24,32,33]. Binding of IFN-γ to its cell surface receptors initiates Janus kinase-mediated phosphorylation of tyrosine 701, which triggers its dimerization, translocation to the nucleus and activation of gene transcription [24]. In addition, phosphorylation of serine 727 is required for maximal activity [24]. To evaluate whether the DGLA-mediated inhibition of IFN-γ induced MCP-1 and ICAM-1 expression involved modulation of phosphorylation at these two sites, Western blot analysis was performed using phospho-specific antibodies. Fig. 2 shows that DGLA inhibits the IFN-γ induced phosphorylation of STAT1 on serine 727 (p = 0.012) without affecting that on tyrosine 701.

**Fig. 3:**
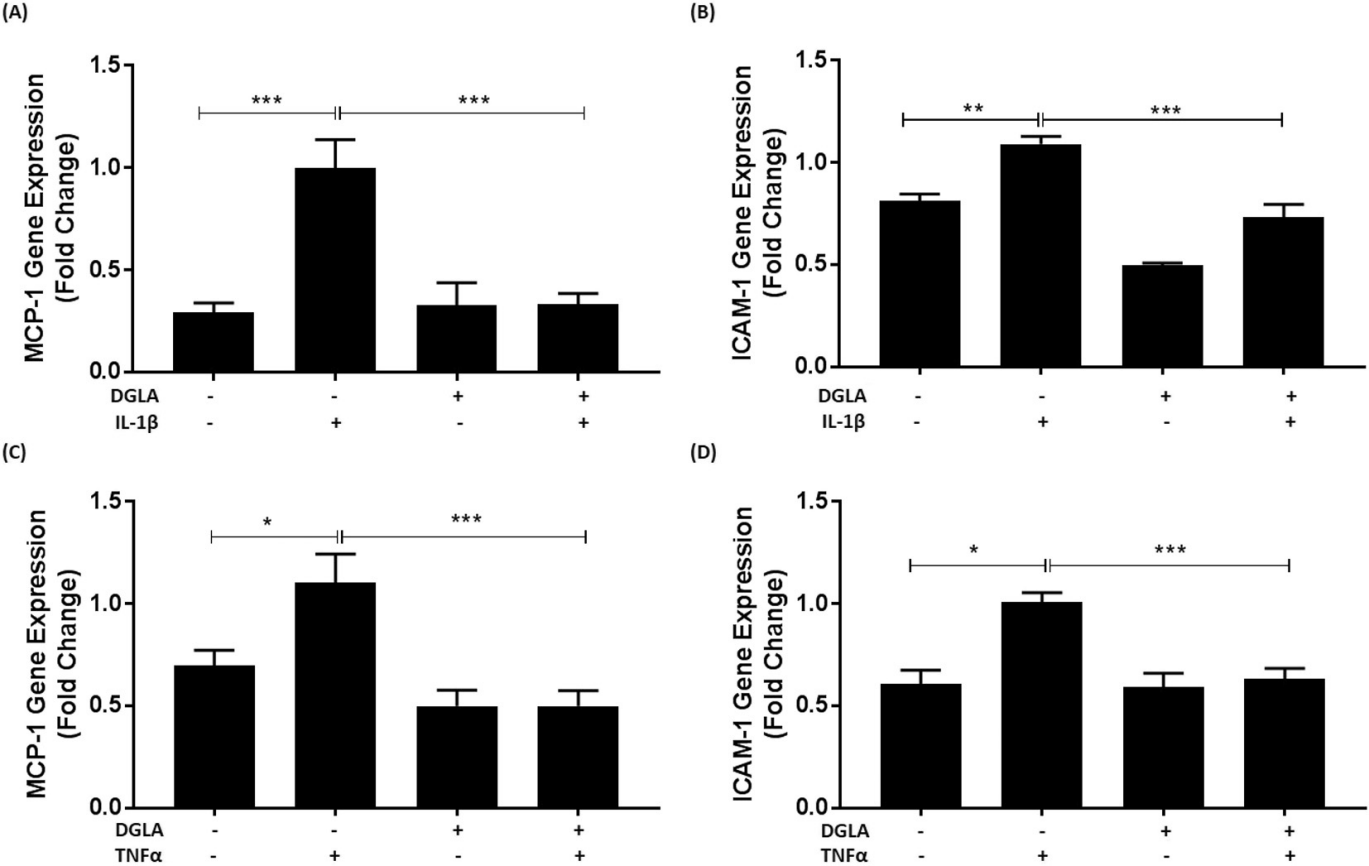
DGLA attenuates IL-1β and TNF-α induced MCP-1 and ICAM-1 expression in human macrophages. THP-1 macrophages were pre-incubated for 24 h with 50 μM DGLA (+) or DMSO vehicle (−). The cells were then treated for 24 h with 1000 U/ml of IL-1β or TNF-α as indicated (+) or its vehicle (−). Total RNA was subjected to RT-qPCR using primers against MCP-1 (A, C), ICAM-1 (B, D) or GAPDH. The mRNA levels were calculated using the comparative Ct method and normalized to the housekeeping gene with values from cells pre-incubated with DMSO vehicle and then treated with the cytokine given an arbitrary value of 1. Graphs display normalized gene expression (mean ± SEM) from three independent experiments. Statistical analysis was performed using a One-way ANOVA (equal variances) with Tukey's post hoc analysis (*, p ≤ 0.05; **, p ≤ 0.01; ***, p ≤ 0.001).

To rule out the possibility that the inhibitory action of DGLA was restricted to IFN-γ, its effect on the induced expression of MCP-1 and ICAM-1 by two other key pro-atherogenic cytokines, IL-1β and TNF-α [5] was determined. Fig. 3 shows that DGLA attenuates the induction of MCP-1 and ICAM-1 expression by both IL-1β (p < 0.001 and p = 0.001 respectively) and TNF-α (p < 0.001 in both cases).

Cholesterol crystal-mediated inflammasome activation and IL-1β production is also involved in chronic inflammation during atherosclerosis [5]. However, consistent with a previous study on mouse macrophages [35], DGLA had no effect on cholesterol crystal-mediated secretion of IL-1β in human macrophages (data not shown). ROS production is also associated with inflammation and oxidation of LDL [3]. The effect of DGLA on tert-butyl hydroperoxide (TBHP)-induced ROS production in both monocytes and macrophages was therefore investigated. Supplementary Fig. 3 shows that DGLA had no effect on ROS levels produced by TBHP in both monocytes and macrophages. In addition, mitochondrial ROS production was not affected by DGLA (Supplementary Fig. 4).

### 3.2 DGLA attenuates chemokine-driven monocytic migration and macrophage foam cell formation

**Fig. 4:**
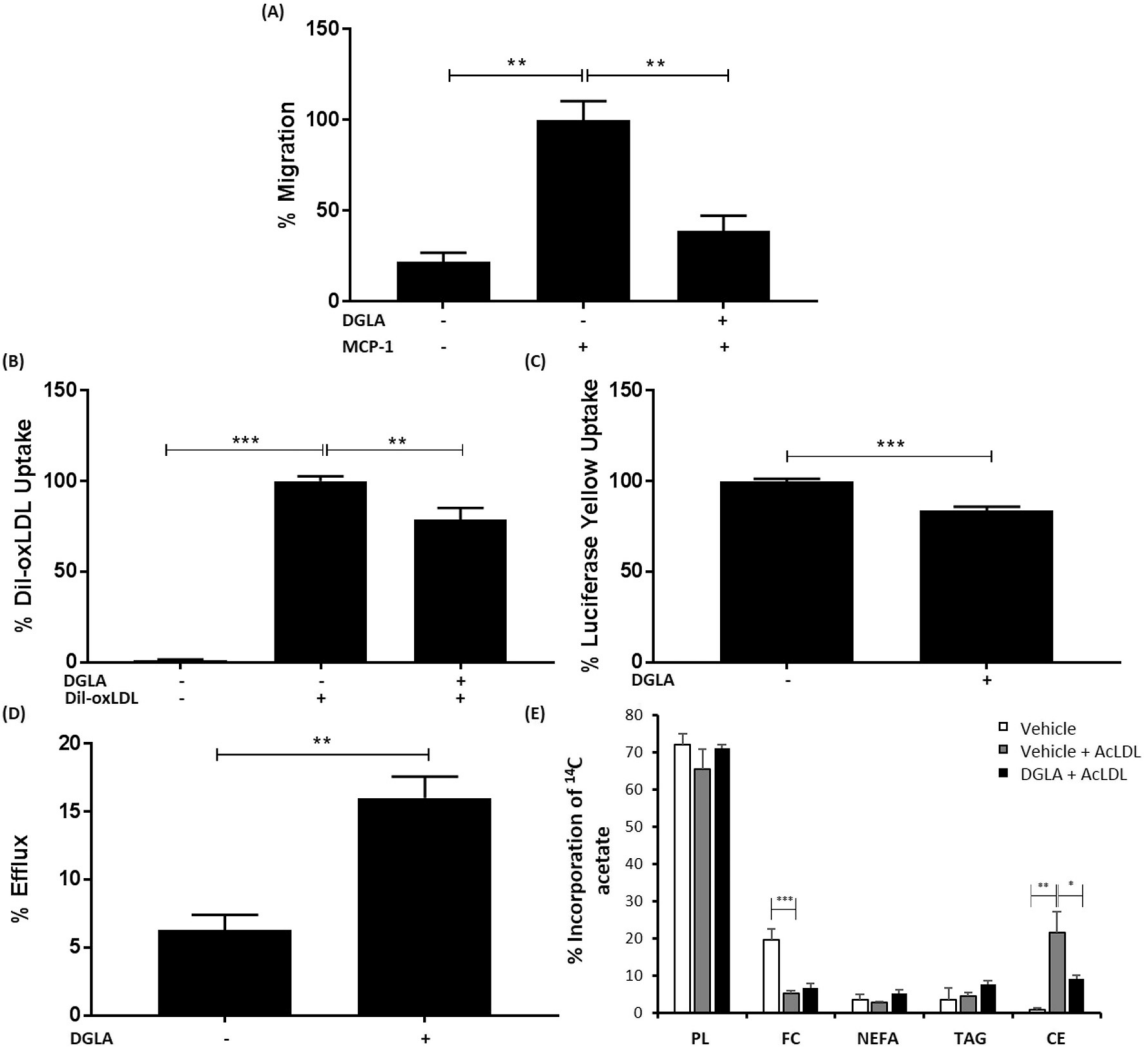
DGLA inhibits MCP-1 driven monocytic migration and macrophage foam cell formation. (A) Migration assays were carried out with THP-1 monocytes incubated for 3 h with 50 μM DGLA (+) or vehicle (−) using MCP-1 (+, 20 ng/ml) as a chemoattractant. Cells incubated with vehicle in the absence MCP-1 were also included for comparative purposes. Monocyte migration was calculated by counting the number of cells that had migrated across a cell insert and expressed as a percentage of total input cells. Graph displays percentage migration (mean ± SEM) from three independent experiments, with values from cells incubated with vehicle and MCP-1 arbitrarily assigned as 100%. (B) THP-1 macrophages were incubated with 50 μM DGLA (+) or vehicle (−) for 24 h and then with Dil-oxLDL (+) for a further 24 h. Cells treated with vehicle in the absence of Dil-oxLDL were also included for comparative purposes. The Dil-oxLDL uptake in cells incubated with vehicle and Dil-oxLDL has been arbitrarily assigned as 100%. The graph indicates the uptake (mean ± SEM) from four independent experiments. (C) THP-1 macrophages were incubated with 50 μM DGLA (+) or vehicle (−) for 24 h and then with LY for a further 24 h. LY uptake in cells incubated with vehicle has been arbitrarily assigned as 100%. The graph indicates the LY uptake (mean ± SEM) from three independent experiments. (D) THP-1 macrophages were pre-treated with 50 μM DGLA (+) or vehicle (−) for 24 h and then incubated for 24 h in the presence of 25 μg/ml AcLDL and 0.5 μCi [^14^C]-cholesterol in RPMI media containing 0.2% (w/v) BSA. Cells were then incubated with 10 μg/ml ApoAI for 24 h. Background cholesterol efflux (in the absence of ApoA1, not displayed on graph) was subtracted from those incubated with ApoA1. The graph shows percentage cholesterol efflux (mean ± SEM) from three independent experiments. (E) RAW264.7 macrophages were pre-treated with 100 μM DGLA or vehicle for 24 h prior to the addition of 50 μM AcLDL and 1 μCi [1-^14^C] acetate for a further 24 h. Cells incubated with vehicle but in the absence of AcLDL were also included for comparative purposes. Lipids were extracted, separated by TLC and radioactive incorporation into individual lipid classes was determined by scintillation counting. Graph shows percentage incorporation of [1-^14^C]-acetate per mg of protein into polar lipids (PL), free cholesterol (FC), non esterified fatty acids (NEFA), triacylglycerols (TAG) and cholesteryl esters (CE). Statistical analysis was performed using either a One-way ANOVA with Tukey's post hoc analysis (A, B, E) or an unpaired Student's *t*-test (C, D) (*, p ≤ 0.05; **, p ≤ 0.01; ***, p ≤ 0.001).

Studies in ApoE^−/−^ mice showed reduced macrophage burden in aortic lesions following DGLA feeding [20]. This could potentially be because of reduced chemokine-driven recruitment of monocytes. To investigate this potential mechanism, the effect of DGLA on monocyte migration induced by the key chemokine, MCP-1, was determined. DGLA inhibits MCP-1 driven migration of monocytes (p = 0.005; Fig. 4A).

Macrophage foam cell formation is dependent on the uptake of modified LDL and the efflux of cholesterol from foam cells [4]. The scavenger receptor-mediated uptake of modified LDL is important in foam cell formation [4]. DGLA significantly attenuates the uptake of Dil-labeled oxLDL (p = 0.003; Fig. 4B). Macropinocytosis is another process involved in the uptake of lipoproteins that contributes to foam cell formation [4,28]. DGLA also attenuates macropinocytosis as judged by the uptake of the dye lucifer yellow (LY) [28] (p < 0.001; Fig. 4C). The effect of DGLA on cholesterol efflux was also monitored by first converting macrophages into foam cells using AcLDL, which is avidly taken up by the cells [22,24,25], in the presence of [^14^C]-cholesterol. The efflux of radiolabeled cholesterol to ApoAI acceptor present in HDL particles was then determined [23,25]. DGLA stimulates the efflux of cholesterol from foam cells (p = 0.008; Fig. 4D).

Foam cell formation is associated with increased intracellular levels of cholesteryl esters [4,25]. We therefore investigated the effect of DGLA on the incorporation of [1-^14^C]-acetate into various lipid classes (polar lipids, free cholesterol, non esterified fatty acids, triacylglycerols and cholesteryl esters) following incubation with AcLDL by TLC and scintillation counting. RAW264.7 macrophages were used for these studies because initial experiments showed that they accumulate higher levels of cholesteryl esters than THP-1 macrophages (data not shown). Preliminary optimization experiments showed that treatment with 100 μM DGLA for 24 h was optimal for analysis of changes in cholesteryl esters levels (data not shown) so these were used for subsequent studies. Fig. 4E shows that the cellular cholesteryl ester content was significantly increased with AcLDL (p = 0.010) and this was attenuated by DGLA (p = 0.032), thereby confirming inhibition of foam cell formation by this fatty acid.

**Fig. 5:**
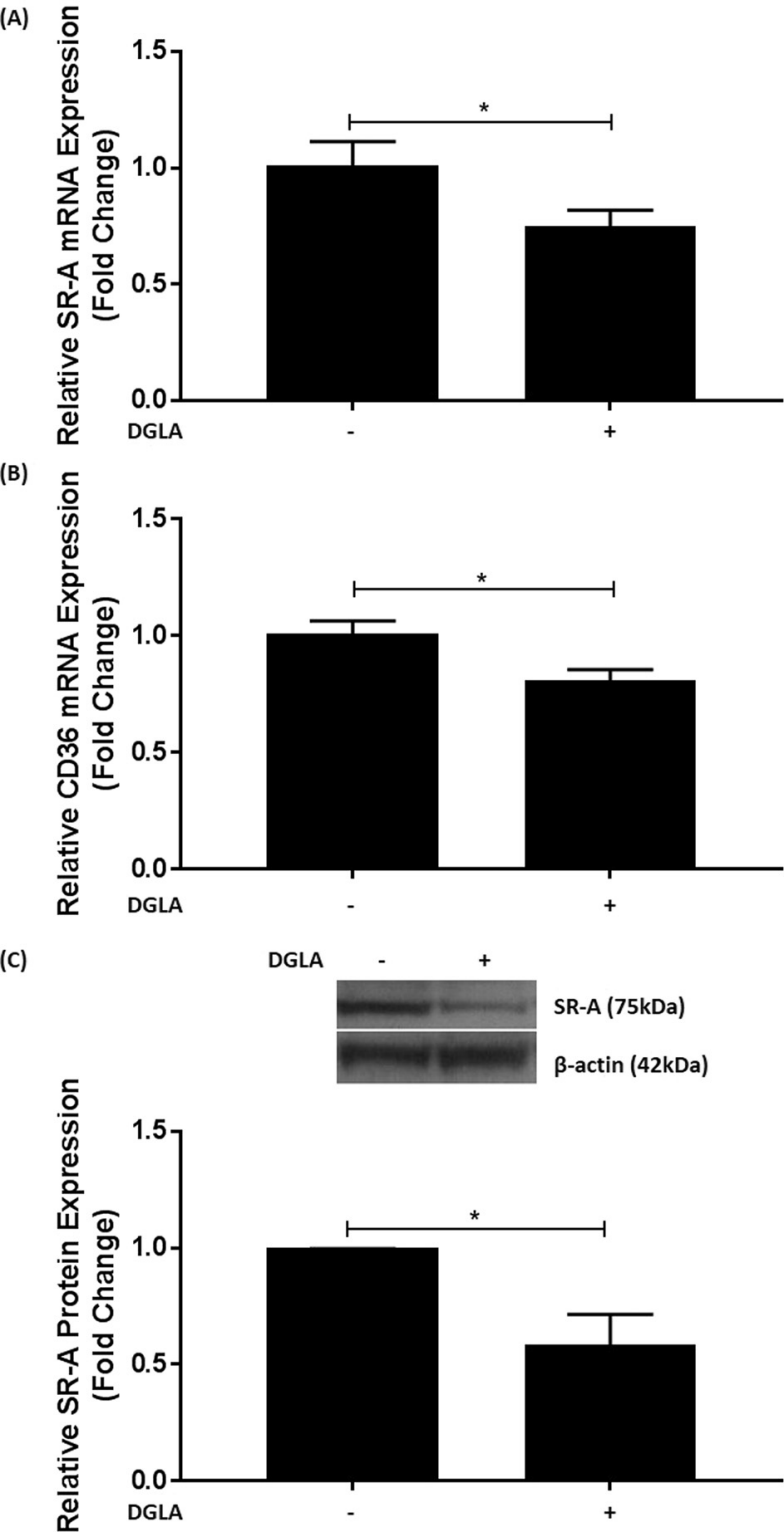
DGLA attenuates the expression of SR-A and CD36 in human macrophages. THP-1 macrophages were pre-incubated for 24 h with 50 μM DGLA (+) or vehicle (−). (A-B) Total RNA was subjected to RT-qPCR using primers against SR-A (A), CD36 (B) or GAPDH. The mRNA levels were calculated using the comparative Ct method and normalized to the housekeeping gene with values from cells treated with vehicle given an arbitrary value of 1. Graphs display normalized gene expression (mean ± SEM) from four independent experiments. (C) Equal amount of proteins were subjected to Western blot analysis with antibodies against SR-A and β-actin. The data were subjected to densitometric and statistical analysis. A representative image is shown on top with graph indicating the relative SR-A expression (mean ± SEM) from four independent experiments. The value from cells treated with vehicle alone has been arbitrarily assigned as 1. In all cases, statistical analysis was performed using an unpaired Student's *t*-test (*, p ≤ 0.05).

Uncontrolled uptake of modified LDL by SRs is crucial in macrophage foam cell formation [4]. The effect of DGLA on the expression of SR-A and CD36, which play crucial roles in modified LDL uptake [4,25], was therefore determined. DGLA attenuates the expression of both these scavenger receptors at the mRNA level (p = 0.047 and p = 0.012 respectively; Fig. 5A–B). To evaluate whether such changes in mRNA expression were followed at the protein level, Western blot analysis was carried out for SR-A. DGLA significantly attenuates SR-A expression (p = 0.019; Fig. 5C). The cell surface expression of SR-A, CD36 and LDL receptor (LDLR) was also determined by flow cytometry and found to be attenuated by DGLA (Supplementary Fig. 5).

### 3.3 DGLA modulates mitochondrial respiration

**Fig. 6:**
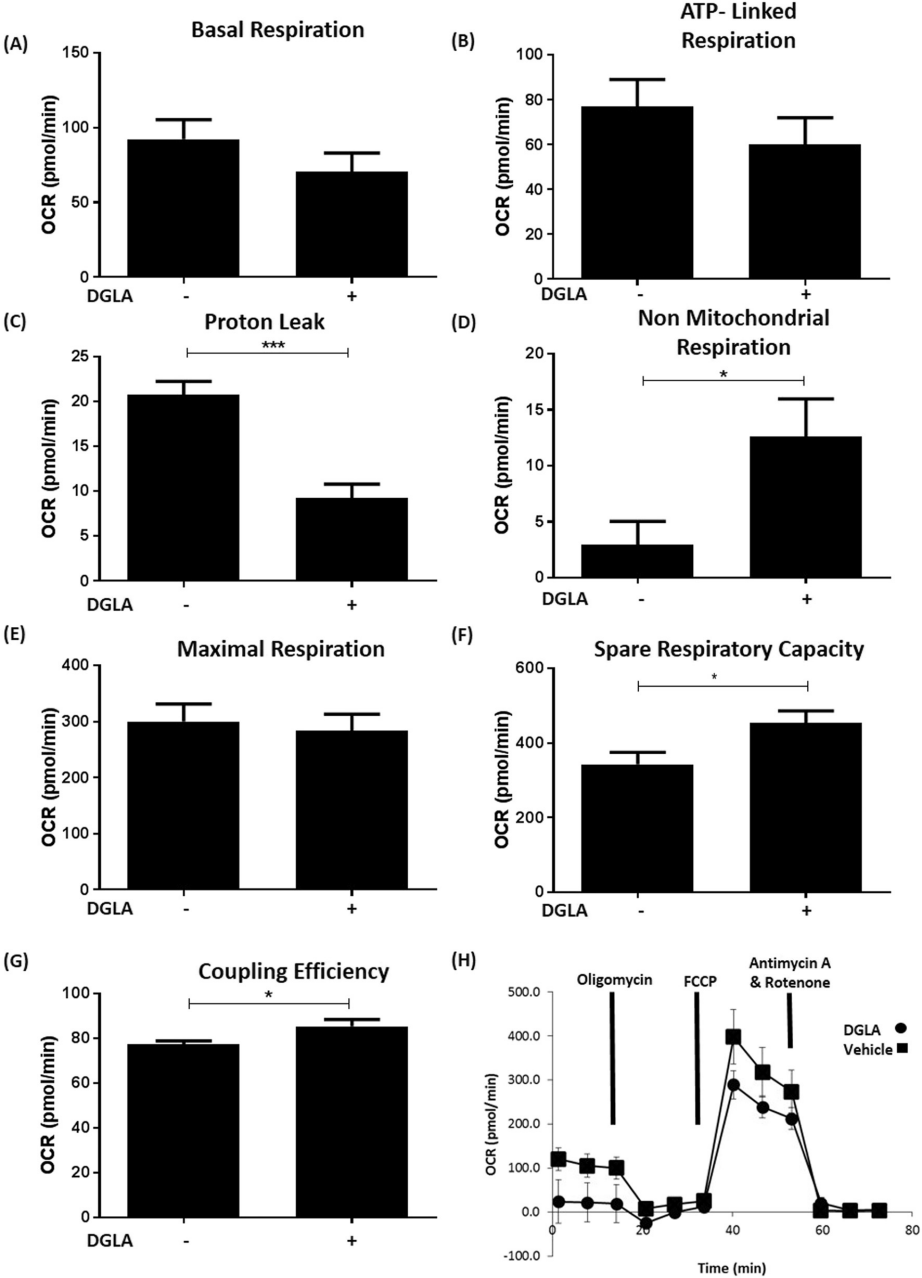
DGLA modulates the bioenergetic profile of human macrophages. THP-1 macrophages were treated with 50 μM DGLA (+) or vehicle (−) for 24 h. Parameters of mitochondrial respiration were analyzed using the Seahorse XF^e^96 analyzer. Readouts included: Basal respiration (A); ATP-linked respiration (B); proton leak (C); non mitochondrial respiration (D); maximal respiration (E); spare respiratory capacity (F) and coupling efficiency (G). Graphs represent mean ± SEM from three independent experiments. Statistical analysis was carried out using an unpaired Student's *t*-test (*, p ≤ 0.05; ***, p ≤ 0.001). A trace from a representative experiment carried out in triplicate is shown in panel H.

Mitochondrial dysfunction has been associated with atherosclerosis [36]. The effect of DGLA at concentration of 50 μM, which has no effect on cell viability or proliferation (see Supplementary Fig. 1), on the bioenergetic profile of THP-1 macrophages was therefore determined using the Seahorse XF^e^96 analyzer. Fig. 6 shows that DGLA produces significant, favourable changes in respiration parameters, including reduction in proton leak (p < 0.001) and increase in non mitochondrial respiration (p = 0.026), spare respiratory capacity (p = 0.026) and coupling efficiency (p = 0.039). No significant changes were seen for basal respiration, ATP-linked respiration and maximal respiration (Fig. 6). Similarly, DGLA produced no significant changes in various parameters related to glycolysis (Supplementary Fig. 6).

### 3.4 GLA is also anti-atherogenic

**Fig. 7:**
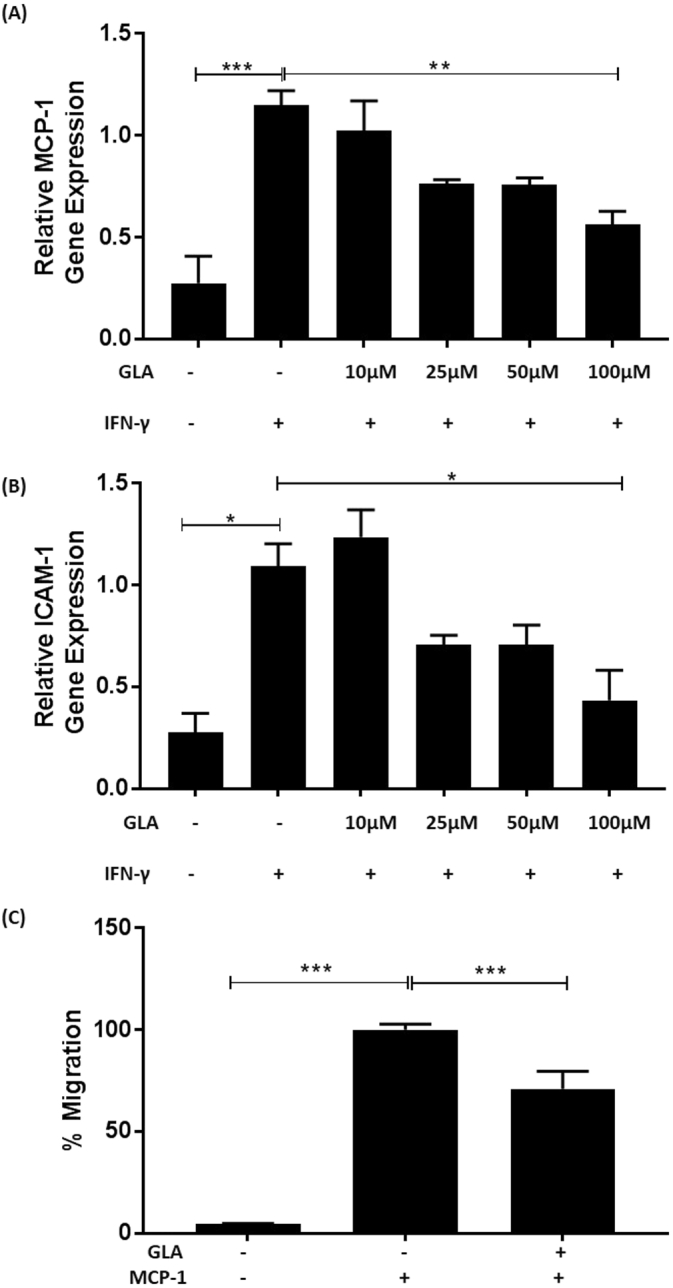
GLA attenuates macrophage pro-inflammatory gene expression and chemokine-driven monocytic migration. (A–B), THP-1 macrophages were pre-incubated for 24 h with the indicated concentration of GLA or DMSO vehicle (−). The cells were then incubated for 3 h with 250 U/ml IFN-γ (+) or its vehicle (−). Total RNA was subjected to RT-qPCR using primers against MCP-1 (A), ICAM-1 (B) or GAPDH. The mRNA levels were calculated using the comparative Ct method and normalized to the housekeeping gene with values from cells pre-incubated with vehicle and then treated with IFN-γ given an arbitrary value of 1. Graphs display normalized gene expression (mean ± SEM) from three independent experiments. (C), migration assays were carried out with THP-1 monocytes incubated for 3 h with 50 μM GLA (+) or vehicle (−) using MCP-1 (20 ng/ml) as the chemoattractant. Cells incubated with vehicle in the absence MCP-1 were also included for comparative purposes. Monocyte migration was calculated by counting the number of cells that had migrated across a cell insert and expressed as a percentage of total input cells. Graph displays percentage migration (mean ± SEM) from three independent experiments, with MCP-1-driven migration in the presence of vehicle arbitrarily assigned as 100%. In all cases, statistical analysis was performed using a One-way ANOVA with Tukey's post hoc analysis (*, p ≤ 0.05; **, p ≤ 0.01; ***, p ≤ 0.001).

It would be expected that GLA, an upstream precursor of DGLA, would also be anti-atherogenic. Representative experiments were therefore performed to investigate this possibility. GLA attenuates IFN-γ induced MCP-1 and ICAM-1 expression at concentrations of 25 μM–100 μM (as used for our previous studies on DGLA shown in Fig. 1A–B) with significant inhibition seen with 100 μM GLA (p = 0.007 and p = 0.011 respectively; Fig. 7A–B). The use of this concentration of GLA also showed inhibition of MCP-1 driven monocytic migration (p < 0.001; Fig. 7C).

### 3.5 PGE1, a key metabolite of DGLA, has similar anti-atherogenic actions

**Fig. 8:**
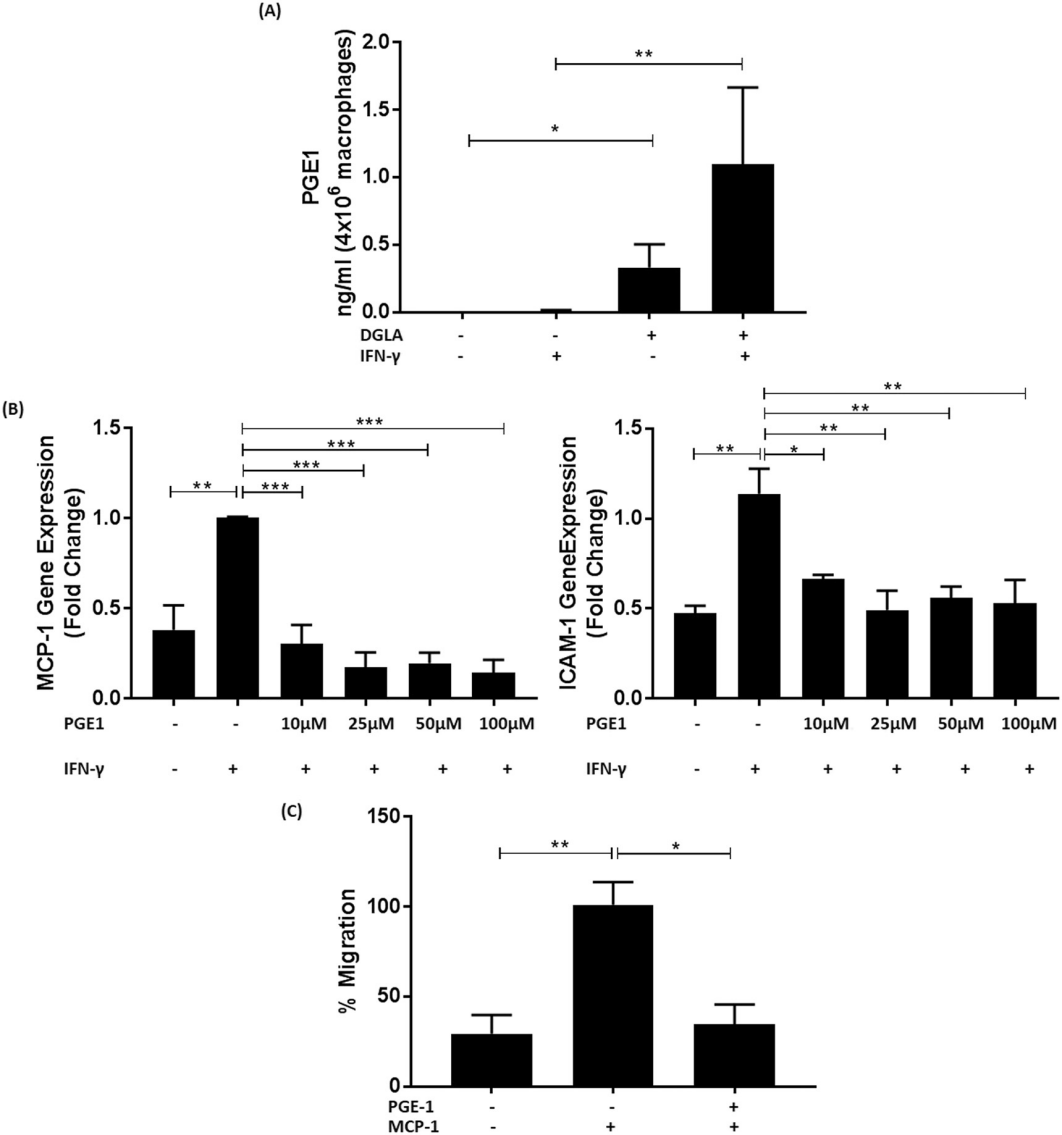
PGE1, a key metabolite of DGLA, inhibits macrophage pro-inflammatory gene expression and chemokine-driven monocytic migration. (A), THP-1 macrophages were pre-incubated for 24 h with 100 μM DGLA (+) or vehicle (−) followed by a further 24 h stimulation with 250 U/ml IFN-γ (+) or its vehicle (−). Media was collected and lipids extracted for measurement using HPLC-MS. Graphs display concentration of PGE1 (ng/ml from 4 × 10^6^ cells) from three independent experiments (mean ± SEM). (B), THP-1 macrophages were pre-incubated for 1 h with the indicated concentration of PGE1 (+) or DMSO vehicle (−). The cells were then treated for 3 h with 250 U/ml IFN-γ (+) or its vehicle (−). Total RNA was subjected to RT-qPCR using primers against MCP-1, ICAM-1 or GAPDH. The mRNA levels were calculated using the comparative Ct method and normalized to the housekeeping gene with values from cells pre-incubated with vehicle and then treated with IFN-γ given an arbitrary value of 1. Graphs display normalized gene expression (mean ± SEM) from three independent experiments. (C), migration assays were carried out with THP-1 monocytes incubated for 3 h with 10 μM PGE1 (+) or vehicle (−) using MCP-1 (20 ng/ml) as the chemoattractant. Cells incubated with vehicle in the absence MCP-1 were also included for comparative purposes. Monocyte migration was calculated by counting the number of cells that had migrated across a cell insert and expressed as a percentage of total input cells. Graph displays percentage migration (mean ± SEM), with MCP-1-driven migration in the presence of vehicle arbitrarily assigned as 100%. Statistical analysis was performed using a Kruskal-Wallis test with Dunn's post hoc test (A) or a One-way ANOVA with Tukey's post hoc analysis (B–C) (*, p ≤ 0.05; **, p ≤ 0.01; ***, p ≤ 0.001).

DGLA can be metabolized to PGE1 via the cyclooxygenase pathway and increased levels of this eicosanoid have been seen following treatment of mouse peritoneal macrophages with GLA [37] and human mononuclear leukocytes with DGLA [38]. In order to confirm that PGE1 levels were indeed increased following stimulation of THP-1 macrophages with DGLA, its concentration in cell supernatants was determined under non-inflammatory and inflammatory conditions, the latter involving stimulation of the cells with the pro-inflammatory cytokine IFN-γ as in the studies shown in Fig. 1, Fig. 2. Thus, the cells were pre-treated for 24 h with the DMSO vehicle or 100 μM DGLA followed by a further 24 h incubation with IFN-γ (250 U/ml) or its vehicle. As shown in Fig. 8A, there was a significant increase in PGE1 levels following pre-treatment of the cells with DGLA both cases (p = 0.024 in the absence of IFN-γ and p = 0.006 in the presence of the cytokine).This suggests that the actions of DGLA could potentially be mediated via PGE1, which would therefore also be expected to have anti-atherogenic actions. In order to investigate this possibility, the effect of several concentrations of PGE1, as our studies on DGLA (Fig. 1A–B) and GLA (Fig. 7A–B), on IFN-γ induced MCP-1 and ICAM-1 expression in THP-1 macrophages was therefore analyzed. A shorter pre-incubation time of 1 h with PGE1 was chosen compared to DGLA because of the short half-life of this prostaglandin [39]. Pre-treatment of the cells with all the concentrations of PGE1 significantly attenuates the IFN-γ induced expression of both MCP-1 and ICAM-1 genes (Fig. 8B; for MCP-1, p = 0.001 for 10 μM PGE1 and p < 0.001 for all the other concentrations and for ICAM-1, p = 0.030 for 10 μM PGE1, p = 0.002 for 25 μM PGE1, p = 0.011 for 50 μM PGE1 and p = 0.004 for 100 μM PGE1). The lowest concentration of PGE1 (10 μM) was used for the analysis of its effects on chemokine driven monocyte migration. Similar to DGLA, PGE1 also significantly attenuates MCP-1 induced monocytic migration (Fig. 8C; p = 0.015).

### 3.6 The action of DGLA extends to endothelial cells and VSMC

**Fig. 9:**
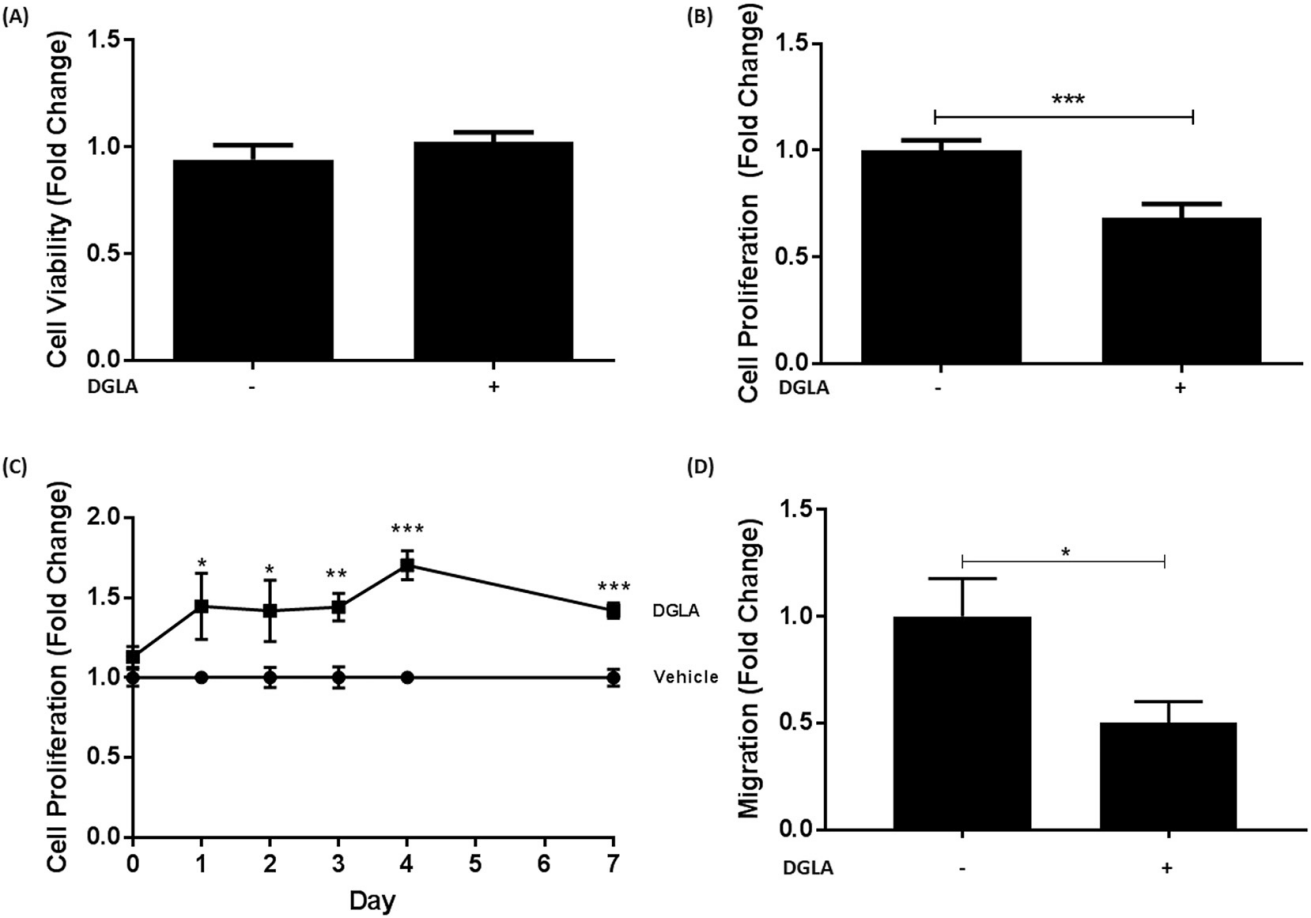
DGLA modulates the properties of endothelial cells and VSMC. (A–B), HUVEC were incubated with 50 μM DGLA (+) or vehicle (−) for 24 h. Media were removed and used to determine LDH release. The remaining cells were then used in the crystal violet assay. Both graphs display fold-change of absorbance values (mean ± SEM) in comparison to vehicle control (arbitrarily assigned as 1) from three independent experiments. (C), The proliferation of HASMC treated with vehicle or 50 μM DGLA over 7 days was carried out by consecutive crystal violet assays between days 0 and 7. Each day had its own vehicle control (arbitrarily assigned as 1) and proliferation was determined relative to this. (D), HASMC migration was carried out as described in Materials and methods. The results are expressed as fold change of migrated cells determined in five fields of view in relation to the vehicle control (−; arbitrarily assigned as 1). Graphs show mean ± SEM from three independent experiments. In all cases, statistical analysis was carried out using an unpaired Student's *t*-test (*, p ≤ 0.05; **, p ≤ 0.01 ***, p ≤ 0.001). For C, similar outcome was obtained by regression analysis.

The effect of DGLA on proliferation of HUVEC was determined because endothelial cell proliferation plays an important role in atherosclerosis [40]. Fig. 9A–B shows that DGLA significantly attenuates proliferation of these cells (p = 0.001) without affecting their viability. In contrast, similar to macrophages (Supplementary Fig. 3), the TBHP-induced production of ROS was not affected in HUVEC (Supplementary Fig. 7).

The effect of DGLA on VSMC was also investigated because previous *in vivo* studies in ApoE^−/−^ mice showed reduced α-smooth muscle cell actin-positive area to the intimal area following feeding of DGLA [20]. DGLA at 50 μM had no effect on the viability of HASMC (data not shown). However, the proliferation of these cells over a 7-day-period was increased by DGLA (Fig. 9C). In contrast, their migration in response to the growth factor PDGF-BB was significantly attenuated (p = 0.031; Fig. 9D) and could account for reduced numbers of smooth muscle cells seen *in vivo* [20].

## 4 Discussion

Omega-6 PUFAs are generally considered pro-atherogenic because of their ability to produce pro-inflammatory lipids and antagonize the actions of omega-3 PUFAs [1,2,9]. However, not all omega-6 PUFAs are pro-atherogenic and previous studies in ApoE^−/−^ mice showed that DGLA supplementation reduced plaque burden and lipid content in the aorta together with levels of macrophages and smooth muscle cells, and the expression of ICAM-1 and VCAM-1 [20]. However, the molecular mechanisms responsible for such beneficial changes are poorly understood and were therefore addressed in this study. DGLA attenuates MCP-1 and ICAM-1 expression in macrophages induced by three key pro-atherogenic cytokines, IFN-γ, IL-1β and TNF-α [5] (Fig. 1, Fig. 3). For IFN-γ, the cytokine-mediated phosphorylation of STAT1 on serine 727 was attenuated by the fatty acid (Fig. 2). DGLA also attenuates MCP-1 driven migration of monocytes (Fig. 4A), a potential mechanism for reduced content of monocytes/macrophages seen in studies on ApoE^−/−^ mice *in vivo* [20]. Consistent with reduced plaque lipid content *in vivo* [20], DGLA inhibits macrophage foam cell formation via reduced uptake of modified LDL, due to decreased expression of key scavenger receptors, and macropinocytosis together with increased cholesterol efflux from foam cells (Fig. 4, Fig. 5). DGLA also produced favourable changes in mitochondrial bioenergetic profile, particularly significant reduction in proton leak (Fig. 6) often associated with mitochondrial dysfunction [41]. GLA, an upstream precursor of DGLA, also attenuates IFN-γ-induced MCP-1 and ICAM-1 expression and chemokine-driven monocytic migration (Fig. 7). PGE1, whose levels increase markedly following pre-treatment of the cells with DGLA, also attenuates the IFN-γ induced expression of MCP-1 and ICAM-1 together with chemokine driven monocytic migration (Fig. 8). The action of DGLA was not restricted to macrophages but extended to endothelial cells and VSMC (Fig. 9). Thus, the DGLA-mediated inhibition of VSMC migration (Fig. 9D) provides a potential mechanism for a decrease in these cells in ApoE^−/−^ mice *in vivo* [20]. These studies also reveal potential cell type specific mechanisms for DGLA actions (e.g. inhibition of endothelial cell proliferation whereas stimulation of this in the case of VSMC; Fig. 9). The mechanisms underlying such differential actions of DGLA remains to be determined. Overall, taken together, the studies presented here provide novel insights into the molecular mechanisms underlying the anti-atherogenic actions of DGLA.

Inflammation in atherosclerosis is orchestrated by cytokines and IFN-γ, IL-1β and TNF-α represent three major pro-atherogenic cytokines [5]. In addition, the importance of IL-1β and the potential of targeting inflammation were revealed by the positive outcome of the CANTOS trial using canakinumab, a therapeutic monoclonal antibody targeting this cytokine [2]. DGLA attenuates the expression of MCP-1 and ICAM-1 induced by three cytokines (Fig. 1, Fig. 3) and this represents a potential mechanism for reduced expression of ICAM-1 (and VCAM-1) in ApoE^−/−^ *in vivo* [20]. Such anti-inflammatory action may potentially extend to other inflammatory disorders. Thus, reduced production of MCP-1 was also seen in inflamed mouse ear produced by croton oil application following oral administration of DGLA-producing *Saccharomyces cerevisiae* [18]. In addition, individuals with arthritis have higher incidence of atherosclerosis and DGLA attenuated IL-1β stimulated proliferation of human adherent synovial cells [42], a hall mark of rheumatoid arthritis. DGLA also attenuated LPS-induced generation of TNF-α in PBMC [31] so the anti-inflammatory actions of this fatty acid extend to the production of cytokines. The CANTOS trial revealed higher incidence of fatal infections [2] so dampening inflammation by multiple cytokines using nutraceuticals such as DGLA might be a safer option than drastic reduction of the action of cytokine(s) via the use of monoclonal antibodies or soluble decoy receptors.

STAT1 is a key transcription factor in IFN-γ signaling [24,32] and is also required for optimal foam cell formation and atherosclerotic lesion development *in vivo* [43]. DGLA inhibits IFN-γ-induced phosphorylation of STAT1 on serine 727 but not tyrosine 701 (Fig. 2), a potential mechanism for attenuation of MCP-1 and ICAM-1 expression induced by this cytokine (Fig. 1). Phosphorylation of STAT1 on serine 727 is required for maximal activity and knock-in mice where this amino acid has been substituted by alanine show reduced expression of IFN-γ induced gene expression in macrophages [32]. In addition, attenuation of STAT1 serine 727 phosphorylation by inhibition of upstream kinases in human macrophages decreased the IFN-γ-induced expression of several genes, including MCP-1 and ICAM-1, together with the uptake of modified lipoproteins [24]. This inhibition of IFN-γ-induced STAT1 serine 727 phosphorylation potentially represents a key anti-atherogenic action of DGLA because this cytokine is known to regulate about 30% of the macrophage transcriptome [32]. In addition to IFN-γ, STAT1 has been implicated as a point of convergence and integration for other pro-inflammatory signaling pathways resulting in increased smooth muscle cell and leukocyte activation and migration [44]. Indeed, reduced atherosclerotic lesion size and progression in diabetic mice following blockade of tumour necrosis factor-like weak inducer of apoptosis (TWEAK/Tnfsf12) was mediated through suppression of STAT1 signaling [45]. It is therefore possible that the DGLA-mediated inhibition of IL-1β- and TNF-α-induced gene expression could also be mediated via STAT1 though other transcription factors such as nuclear factor-κB [3] may also be involved.

Omega-3 PUFAs appear to have some similar anti-atherogenic actions as DGLA reported here. For example, our previous studies have shown that eicosapentaenoic acid (EPA) and docosahexaenoic acid (DHA) inhibited MCP-1-driven monocytic migration [23] and macropinocytosis [46]. However, some differences in the mechanisms of action have also been identified. Thus, EPA and DHA, but not DGLA, attenuated inflammasome activation [35]. In addition, DGLA attenuated the expression of SR-A and CD36 (Fig. 5) whereas this was not seen with EPA and DHA [46].

Foam cell formation, a critical early event in atherosclerosis [4], is a complex process involving chemokine-driven recruitment of monocytes and their differentiation to macrophages, production of ROS leading to the oxidation of LDL, uptake of such oxLDL by macrophages, and the efflux of cholesterol from foam cells to acceptors such as HDL or its key apolipoprotein ApoA1 and subsequent reverse cholesterol transport [4]. Although DGLA had no effect on ROS production (Supplementary Figs. 3 and 7), the fatty acid attenuates chemokine-driven monocytic migration together with the uptake of modified LDL by both SR-mediated endocytosis and macropinocytosis, and stimulates cholesterol efflux from foam cells (Fig. 4). Such an anti-foam activity is likely to be a major contributor to the anti-atherogenic actions of DGLA and potentially responsible for the reduced plaque lipid content in ApoE mice *in vivo* [20]. SR-A and CD36 are two key SRs in the receptor-mediated uptake of modified LDL [3,4] and DGLA attenuates the expression of both these SRs (Fig. 5 and Supplementary Fig. 5), potentially a key mechanism for its anti-foam cell action.

The mechanisms underlying the induction of macrophage cholesterol efflux by DGLA remains to be determined. ATP-binding cassette transporter (ABC)-A1 and -G1 are key mediators of macrophage cholesterol efflux to lipid free ApoA1 and HDL respectively and ApoE also contributes to the process [4]. Preliminary experiments showed that DGLA did not increase the expression of ABCA1 and ABCG1 mRNA together with the ABCA1 and ApoE proteins in foam cells (data not shown). ABCA1, ABCG1 and ApoE are regulated by liver X receptors (LXRs) [47]. However, RT-qPCR showed that rather than inducing, DGLA significantly inhibited LXR-α and -β mRNA expression in THP-1 macrophages (Supplementary Fig. 8). Interestingly, inhibition of LXR actions by other beneficial PUFAs such as EPA and linoleic acid (LA) have also been previously demonstrated [[48], [49], [50]]. This suggests mechanisms independent of the LXR/ABCA1/ABCG1/ApoE axis in the promotion of cholesterol efflux by DGLA, similar to some other PUFAs [51,52]. For example, LA stimulated cholesterol efflux in human macrophages and this was associated with increased expression of Cell death-inducing DFF45 like effector and Perilipin-Adipophilin-TIP47 family members [51]. In addition, stimulation of cholesterol efflux by α-linolenic acid was associated with decreased expression of stearoyl CoA desaturase 1, a rate limiting enzyme in the synthesis of monounsaturated fatty acids [52].

## 5 Conclusion

This is the first study that provides detailed mechanistic insight into the anti-atherogenic action of DGLA previously seen in a limited study on ApoE deficient mice *in vivo* [20]. DGLA attenuated several pro-atherogenic processes: pro-inflammatory gene expression by three key cytokines; chemokine-driven monocytic migration; foam cell formation; and VSMC migration. The anti-inflammatory action of DGLA on IFN-γ signaling was via modulation of STAT1 serine 727 phosphorylation. DGLA inhibited foam cell formation by reducing the uptake of modified LDL by macropinocytosis and by decreasing the expression of two key SRs, SR-A and CD36, and stimulated cholesterol efflux from foam cells. DGLA also attenuated proliferation of endothelial cells, and improved mitrochondrial function (i.e. reduced proton leak). These studies highlight the potential of DGLA in the prevention and treatment of atherosclerosis. Future studies should investigate the efficacy of DGLA to cause regression of existing atherosclerotic plaques in mouse model systems, and to reduce CVD burden and associated risk factors in clinical trials.

## Author contributions

HG, JOW, AI, Y-HC, DRM, IAG, VJT, VBO, JLH, IK-G, SB and DPR were responsible for the design of the experiments. Experiments were performed by HG, JOW, NF, AI, Y-HC, DRM, IAG and VJT. Data analysis was performed by HG, JOW, NF, AI, Y-HC, IAG and VJT. HG and JOW prepared the figures and HG and DPR wrote the manuscript. All the authors contributed to the review of the manuscript.

## Transparency document

Transparency document. Image 1

## Appendix A Supplementary data

Supplementary material Image 2
