# Supplementary material for: Dihomo-γ-linolenic acid inhibits several key cellular processes associated with atherosclerosis
Source: Biochim Biophys Acta Mol Basis Dis. 2019 Sep 1;1865(9):2538–50. doi: 10.1016/j.bbadis.2019.06.011 (PMC6620504; doi:10.1016/j.bbadis.2019.06.011)
Supplement: Supplementary file 2 — Supplementary material [file mmc2.docx]

**Supplementary Data**

**Dihomo-γ-linolenic acid inhibits several key cellular processes associated with atherosclerosis**

Hayley Gallagher^1^, Jessica O. Williams^1^, Nele Ferekidis^1^, Alaa Ismail^1^, Yee-Hung Chan^1^, Daryn R. Michael^1^, Irina A. Guschina^1^, Victoria J. Tyrrell^2^, Valerie B. O’Donnell^2^, John L. Harwood^1^, Inna Khozin-Goldberg^3^, Sammy Boussiba^3^ and Dipak P. Ramji^1*^.

^1^Cardiff School of Biosciences, Cardiff University, Sir Martin Evans Building, Museum Avenue, Cardiff CF10 3AX, UK.

^2^Systems Immunity Research Institute, School of Medicine, Cardiff University, Cardiff CF14 4XN, UK.

^3^Microalgal Biotechnology Laboratory, French Associates Institute for Agriculture and Biotechnology of Drylands, J. Blaustein Institutes for Desert Research, Ben-Gurion University of the Negev, Sede Boqer Campus, 84990, Israel.

***Corresponding author:** Professor Dipak P. Ramji, Cardiff School of Biosciences, Cardiff University, Sir Martin Evans Building, Museum Avenue, Cardiff, CF10 3AX, UK. Tel: 0044 (0)29 20876753; Fax: 0044 (0)29 20874116; Email: Ramji@Cardiff.ac.uk

**Supplementary Table 1. Sequences of primers used for RT-qPCR.**

| **Gene** | **Forward Primer Sequence**  **(5’ to 3’)** | **Reverse Primer Sequence**  **(5’ to 3’)** |
| --- | --- | --- |
| hGAPDH | CTTTTGCGTCGCCAGCCGAG | GCCCAATACGACCAAATCCGTTGACT |
| hMCP-1 | CGCTCAGCCAGATGCAATCAATG | ATGGTCTTGAAGATCACAGCTTCTTTGG |
| mMCP-1 | GCTCAGCCAGATGCAGTTAACG | GCTTGGTGACAAAAACTACAGCTTC |
| hICAM -1 | GACCAGAGGTTGAACCCCAC | GCGCCGGAAAGCTGTAGAT |
| mICAM-1 | ACGTGCTGTATGGTCCTCGG | GTCCAGTTATTTTGAGAGTGGTACAGTA |
| hSR-A | GTCCAATAGGTCCTCCGGGT | CCCACCGACCAGTCGAAC |
| hCD36 | AGCCATTTTAAAGATAGCTTTCC | AAGCTCTGGTTCTTATTCACA |
| mβ-actin | ACACCCGCCACCAGTTCGCCAT | CACACCCTGGTGCCTAGGGCGGCCCACGATC |
| hLXR-α | \| CCTTCAGAACCCACAGAGATCC \| \| --- \| | \| ACGCTGCATAGCTCGTTCC \| \| --- \| |
| hLXR-β | \| GCTAACAGCGGCTCAAGAACT \| \| --- \| | \| GGAGCGTTTGTTGCACTGC \| \| --- \| |

CD36, cluster of differentiation 36; GAPDH, glyceraldhehyde 3-phosphate dehydrogenase; h, human; ICAM-1, intercellular adhesion molecule-1; LXR, liver X receptors; m, mouse, MCP-1, monocyte chemotactic protein-1, SR-A, scavenger receptor A

**
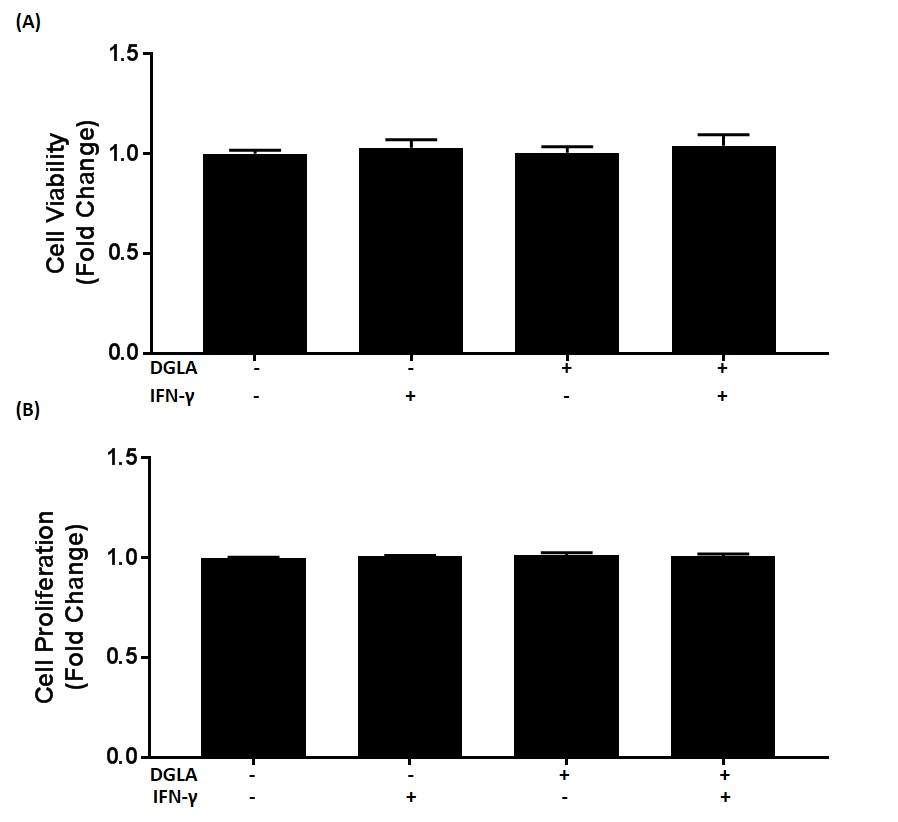
Supplementary Figure 1. DGLA has no effect on viability or proliferation of human macrophages**

THP-1 macrophages were incubated with 50 μM DGLA (+) or DMSO vehicle (-) for 24 h and then in the presence of 250 U IFN-γ (+) or its vehicle (-) for a further 3 h. Media was removed and used to determine LDH release. The remaining cells were then used for the crystal violet assay. The graphs display fold-change of absorbance values (mean +/- SEM) in comparison to the vehicle alone (arbitrarily assigned as 1) from three independent experiments. Statistical analysis was performed using One-way ANOVA with Tukey’s post hoc test.

**
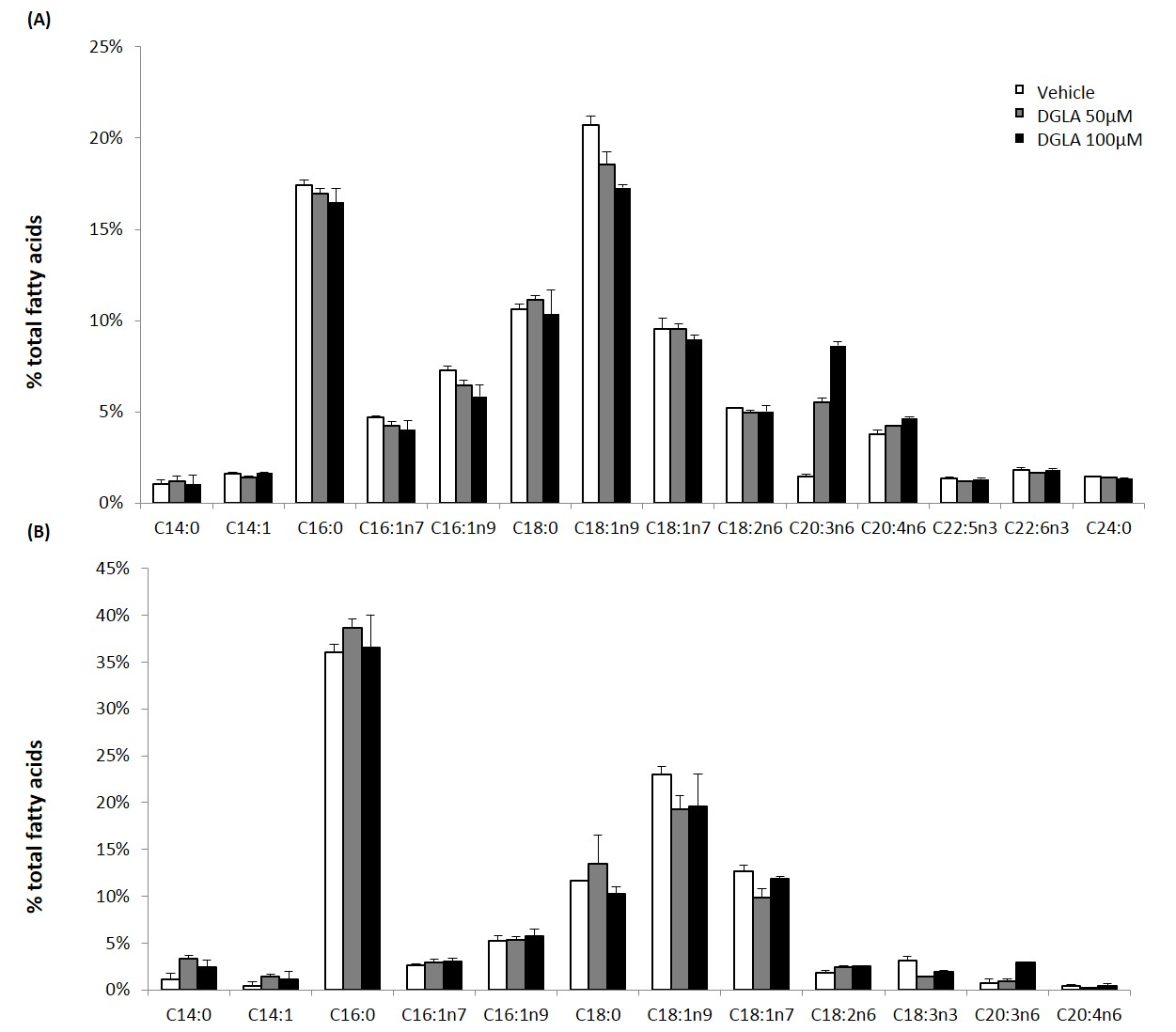
**

**Supplementary Figure 2. DGLA was taken up by macrophages and incorporated into total polar lipids and triacylglycerol fractions**

THP-1 macrophages were incubated with 50 μM or 100 μM of DGLA or the DMSO vehicle for 24 h. Lipids were extracted and separated by thin layer chromatography and the fatty acid profiles in total polar lipids and total triacylglycerol fractions (A and B, respectively) were determined by gas liquid chromatography. Graphs display average fatty acid composition of fraction as a percentage (+/- SEM) of one experiment carried out in duplicate. C14:0, myristic acid; C14:1, myristoleic acid; C16:0, palmitic acid; C16:1n7, palmitoleic acid; C18:0, stearic acid; C18:1n9, oleic acid; C18:1n7, *cis*-vaccenic acid; C18:2n6, linoleic acid; C18:3n3, α-linolenic acid; C20:3n6, dihomo-γ-linolenic acid; C20:4n6, arachidonic acid; C22:5n3, docosapentaenoic acid; C22:6n3, docosahexaenoic acid; C24:0, lignoceric acid.

**
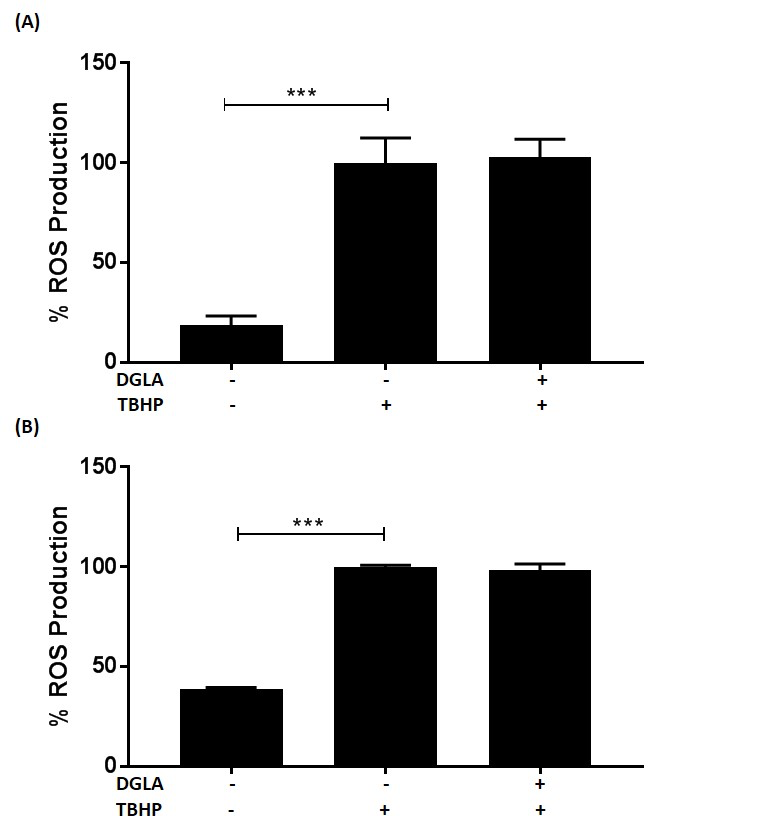
**

**Supplementary Figure 3. DGLA has no effect on TBHP-induced ROS production in human monocytes and macrophages**

THP-1 monocytes (A) were incubated with 35 μM 2’,7’ –dichlorofluorescin diacetate (DCFDA) for 30 min followed by 100 μM TBHP (+) in the presence of 50 μM DGLA (+) or vehicle (-) for 3 h. THP-1 macrophages (B) were pre incubated with 50 μM DGLA (+) or vehicle (-) for 24 h prior to incubation with 35 μM DCFDA for 30 min and 100 μM TBHP for 3 h. In both cases, cells incubated with vehicle in the absence of TBHP were also included for comparative purposes. Fluorescence was measured at 495 nm and 529 nm for excitation and emission spectra respectively. Graph displays mean +/- SEM from three independent experiments. The value in cells treated with vehicle and TBHP has been arbitrarily assigned as 100%. Statistical analysis was performed using a One-way ANOVA followed by Tukey’s post hoc analysis (***, P ≤ 0.001).

**
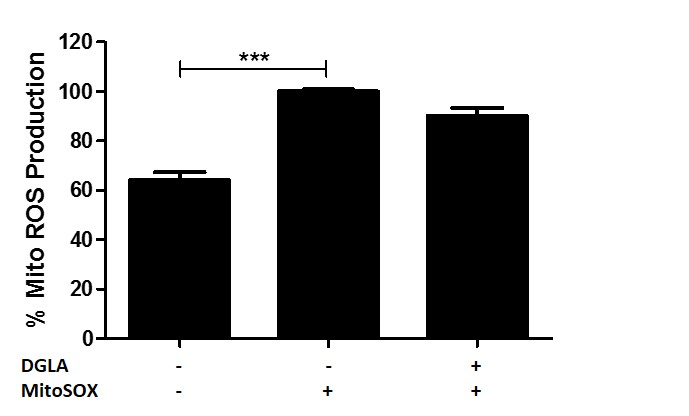
**

**Supplementary Figure 4. DGLA has no effect on mitochondrial ROS production in human macrophages**

THP-1 macrophages were incubated for 24 h with 50 μM DGLA (+) or vehicle (-) followed by 5 μM MitoSOX (+) for 30 min. Cells incubated with vehicle in the absence of MitoSOX were also included for comparative purposes. Fluorescence was measured at 510 nm and 580 nm for excitation and emission spectra respectively. Graph displays mean +/- SEM from four independent experiments. The value in cells treated with vehicle and MitoSOX has been arbitrarily assigned as 100%. Statistical analysis was performed using a One-way ANOVA followed by Tukey’s post hoc analysis (***, P ≤ 0.001).


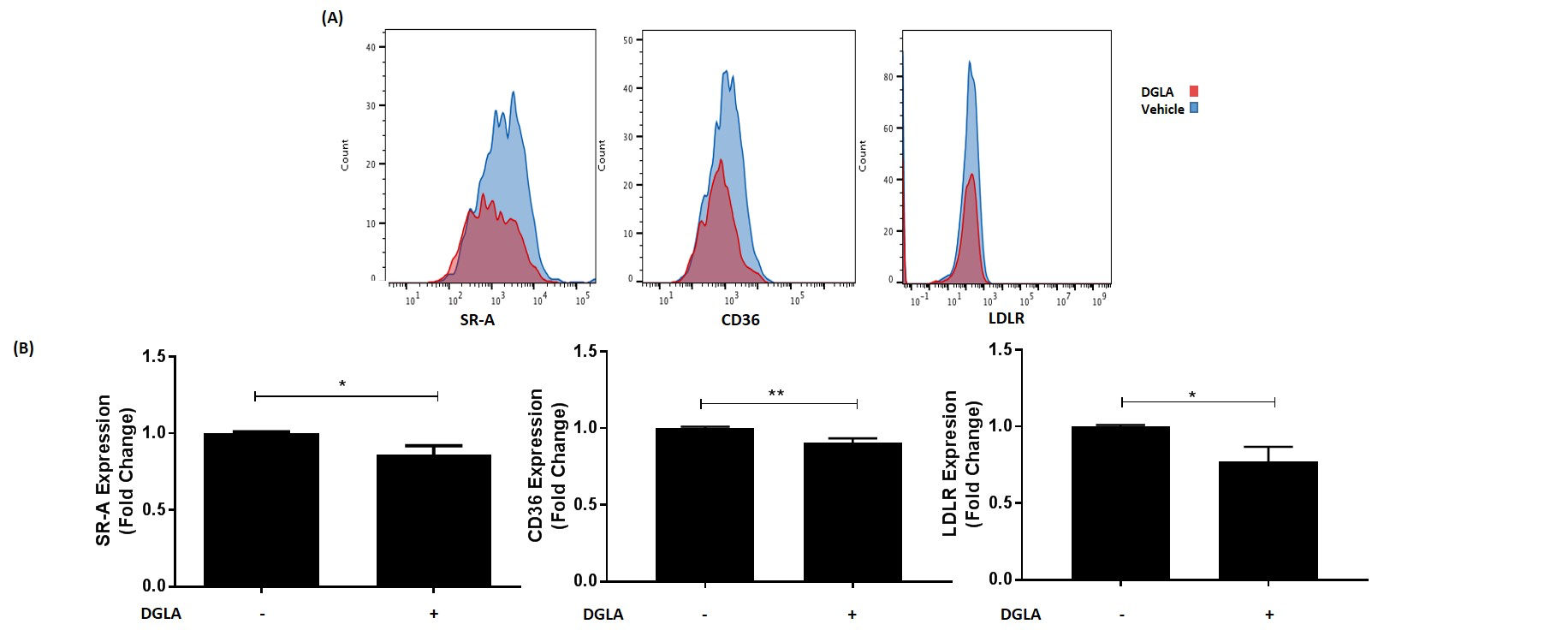


**Supplementary Figure 5. DGLA attenuates the cell surface expression of SR-A, CD36 and LDLR**

THP-1 macrophages were incubated for 24 h with vehicle (-) or 50 μM DGLA (+). The cell surface expression of SR-A, CD36 and LDLR was determined by flow cytometry. (A), Representative data from flow cytometry; (B), graphs showing mean expression +/- SEM from four independent experiments (levels in cells treated with vehicle has been arbitrarily assigned as 1). Statistical analysis was performed using an unpaired Student’s t test (*, *p* ≤0.05; **, P ≤ 0.01).


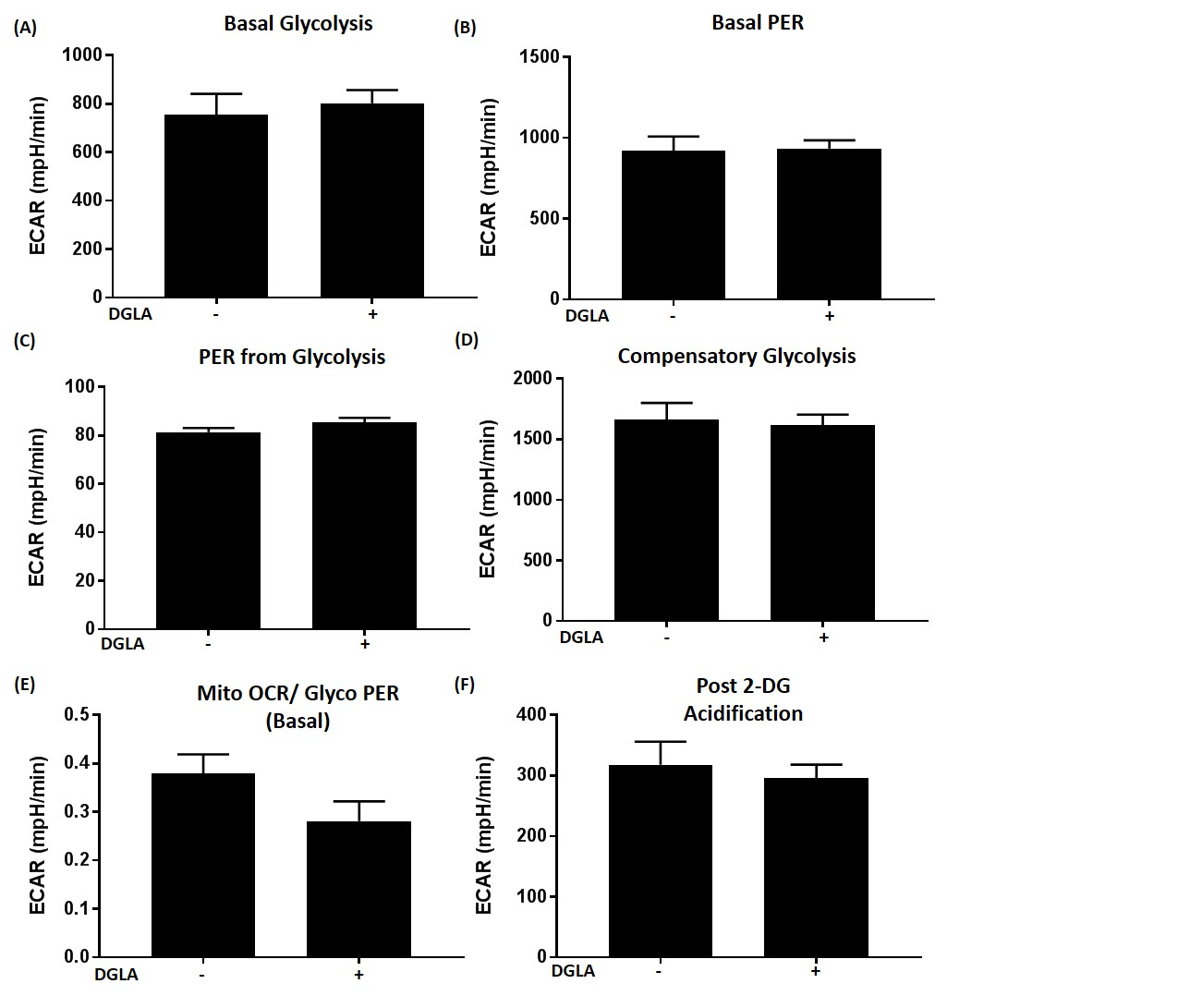


**Supplementary Figure 6. DGLA has no effect on glycolysis in human macrophages**

THP-1 macrophages were treated with 50 μM DGLA (+) or vehicle (-) for 24 h. Parameters of glycolysis were analyzed using the Seahorse XF^e^96 analyzer. Readouts include: Basal Glycolysis (A), Basal Proton Efflux Rate (PER) (B), PER from Basal Glycolysis (C), Compensatory Glycolysis (D), Mitochondrial Oxygen Consumption Rate (OCR)/ Glycolysis PER (E) and Post 2-deoxy-D-glucose (2-DG) Acidification (F). Graphs represent mean +/- SEM from three independent experiments. Statistical analysis was carried out using an unpaired student’s t-test. ECAR, extracellular acidification rate.

**
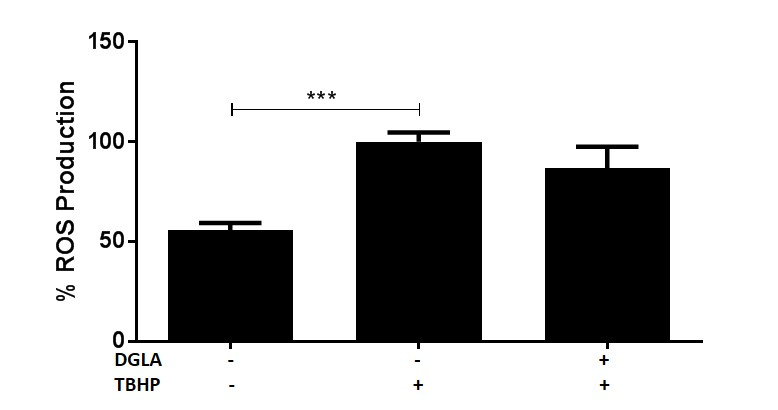
**

**Supplementary Figure 7. DGLA has no effect on ROS production in endothelial cells**

HUVEC were pre incubated with 50 μM DGLA (+) or vehicle (-) for 24 h prior to incubation with 35 μM DCFDA for 30 min and 100 μM TBHP (+) for 3 h. Cells incubated with vehicle in the absence of TBHP were also included for comparative purposes. Fluorescence was measured at 495 nm and 529 nm for excitation and emission spectra respectively. Graph displays mean +/- SEM from three independent experiments. The value in cells treated with vehicle and TBHP has been arbitrarily assigned as 100%. Statistical analysis was performed using a One-way ANOVA followed by Tukey’s post hoc test (***, P ≤ 0.001).

**
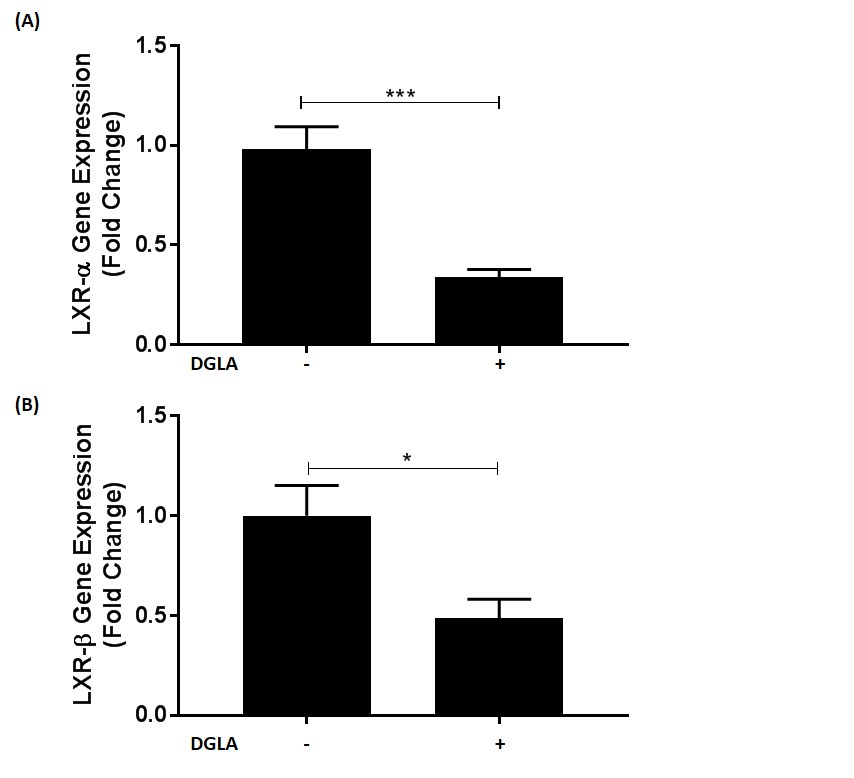
**

**Supplementary Figure 8. DGLA significantly inhibits LXR-α and -β mRNA expression in THP-1 macrophages**

THP-1 macrophages were incubated for 24 h with 50 μM DGLA (+) or vehicle (-). Total RNA was subjected to reverse transcription and RT-qPCR with primers specific for human LXR-α or LXR-β or GAPDH. Graphs display mean gene expression (mean +/- SEM) from three independent experiments with values from vehicle treated cells arbitrarily assigned as 1. Statistical analysis was performed using an unpaired Students t- test (* P ≤0.05, *** P ≤ 0.001).

**Supplementary Methods**

**Reactive oxygen species (ROS) Production**

Production of ROS *in vitro* was determined using a 2’7’-dichlorofluorescin diacetate (DCFDA) Cellular ROS Detection Assay Kit (ab113851) according to the manufacturer’s instructions (Abcam). Tert-butyl hydroperoxide (TBHP) was used as a positive control for ROS production in the cells.

**Analysis of cellular lipids**

Extraction of lipids and separation by one dimension thin layer chromatography (TLC) was carried out as described in the text of the manuscript. For the separation of individual polar lipids, two dimension TLC was carried out using boric acid impregnated silica gel G plates [0.2% boric acid in water: ethanol (1:2, v/v)] with chloroform: methanol: ammonium hydroxide (65:25:4, v/v/v) as a solvent for the 1st dimension and n-butanol: acetic acid: water (90:20:20, v/v/v) for the 2nd dimension. After drying, the plates were sprayed with a 0.05% (w/v) solution of 8- anilino-4-naphthalenesulphonic acid (ANSA) in methanol and lipids were visualized under UV light. The separated lipids were scraped from the plate and subjected to fatty acid analysis by gas liquid chromatography (GLC).

For GLC, the lipids separated by TLC were used for fatty acid methyl esters (FAMEs) preparation. FAMEs were prepared using a solution of 2.5% (v/v) H_2_SO_4_ in dry methanol: toluene (2:1, v/v) for 2 h at 70^0^C. The FAMEs were extracted by two additions of 3 ml of hexane. The combined hexane fractions were dried under a stream of nitrogen and reconstituted in 50 μl of hexane. A known amount of an internal standard, heptadecanoic acid (C17:0), was added to each sample for fatty acid and lipid quantification. FAMEs were analyzed using a Clarus 500 GC with a flame ionisation detector (Perkin-Elmer) and equipped with 30 m by 0.25 mm i. d. capillary column (Elite 225, Perkin-Elmer). The oven temperature program was set to 170^0^C for 3 min, 4^0^C per /min to 220^0^C and final hold for 30 min at 220^0^C. FAMEs were identified by comparison of the peak retention times with those of GC-411 fatty acid standard mixture (Nu-Chek Prep, Inc). TotalChrom software from Perkin-Elmer was used for data acquisition and analysis.

**Determination of cellular glycolysis**

The analysis was carried out using XF^e^96 Seahorse analyzer and XF glycolytic rate assay kit (glycolysis) together with XF-FluxPaks containing 96-well plates and cartridges (Agilent Technologies). The assay kit uses rotenone/antimycin A mix (blocks mitochondrial activity) and 2-deoxy-D-glucose (2-DG; inhibits glycolysis) together with phenol red-free base medium and 5 mM Hepes to determine glycolysis, basal proton efflux rate (PER), glycolytic PER, compensatory glycolysis, extracellular acifidification rate (ECAR) and mitochondrial oxygen consumption rate (OCR): glycolytic PER ratio. Optimisation experiments with a different cell seeding number and titration of various inhibitors was first carried out. These were found to be 200,000 cells per well of a 96-well Seahorse plate and 0.5 µM rotenone/antimycin-A mix and 50 mM 2-DG. The experiments and data analysis was carried out essentially as described by the manufacturer (Agilent Technologies).

**Determination of mitochondrial ROS production**

THP-1 macrophages were pre-treated with vehicle or 50 μM DGLA for 24 h and then stained with 5 μM mitoSOX for 30 min. Fluorescence was then measured at Ex510/Em580 according to manufacturer's instructions (Invitrogen).

**Analaysis of cell surface expression by flow cytometry**

THP-1 monocytes (1 x 10^6^ cells) were differentiated into macrophages and then treated with DGLA or vehicle for 24 h. The cells were then detached using 0.5 ml Trypsin-EDTA (0.05%, w/v) by incubation for 30 min. Then, 0.5 ml of complete culture medium was added to inactivate the enzyme. The resulting mixture was then pipetted up and down several times, subjected to centrifugation at 9,000 x g for 5 min and re-suspended in 2% (w/v) paraformaldehyde. The cells were stained at 4°C for 30 min with cell surface marker antibodies: PE mouse anti-human MSR1, FITC mouse anti-human CD36 clone (CLB-IVC7) and anti-human LDLR APC Conjugated Mouse IgG1 (BD Biosciences). The cells were then washed with 2% (v/v) PBS and analyzed by flow cytometry using BD LSR Fortessa 4 lasers flow cytometer.
